# Heuristic platelet issuing policies for an integrated ABO blood bank system under heterogeneous demand: towards patient-centric management

**DOI:** 10.3389/frhs.2026.1813185

**Published:** 2026-06-10

**Authors:** Chithraponnu R, Umamaheswari S

**Affiliations:** Department of Mathematics, School of Advanced Sciences, Vellore Institute of Technology (VIT), Chennai, Tamil Nadu, India

**Keywords:** arena, healthcare, issuing policy, patient-centric demand, platelet inventory management, simulation, transfusion-transmitted reactions

## Abstract

**Background:**

Transfusion-transmitted reactions remain a significant concern in platelet transfusion medicine because of inadequate platelet inventory management (PIM) and allocation processes. Existing issuing policies lack structured, patient-centric approaches that adequately address compatibility factors and the allocation of platelet components based on specific treatment demands.

**Methods:**

This model proposes a simulation-based, patient-centric platelet issuing policy that integrates product age preferences, ABO/Rh factor compatibility, and the blood group subtypes of A, and prioritizes platelet product preferences. A hybrid strategy combining Fresh Unit First Use (FUFU) and Old Unit First Use (OUFU) policies ensures precise allocation. Moreover, compatible substitution enables single-donor platelets (SDP) to replace random-donor platelets (RDP) and vice versa for the heterogeneous demand. Model parameters are derived from the literature and supplemented by preliminary expert input. The proposed model was evaluated through case studies, numerical example using MATLAB, and simulation experiments using Arena.

**Results:**

The simulation results show improved operational performance in terms of service level, backlog reduction, and allocation efficiency across multiple demand scenarios. In particular, the conventional ABO system achieved service levels of 0% for the scenario service level of 99.9%, whereas the proposed policy achieved service levels above 50% for the same scenario, demonstrating its effectiveness in handling subtype-specific inventory management.

**Conclusion:**

The proposed heuristic model provides a structured, simulation-based approach for evaluating issuing policies under compatibility and demand conditions. These findings highlight the potential incorporation of subtype classification and hybrid allocation strategies to enhance the operational efficiency of platelet inventory systems. However, the results are derived from a simulation environment, and further real-world validation is required to assess clinical applicability and safety.

**Sustainability development goals 2023:**

^3^Good health and well-being.

## 1 Introduction

Blood can be obtained in various forms, including whole blood units, via mechanical separation to isolate specific components such as red blood cells (RBCs), platelet apheresis, platelet concentrate, and plasma. Among these components, platelets are particularly important because of their limited shelf life of 5–7 days (1–4). Compared with other blood components, platelets have a shorter lifespan but are irreplaceable in clinical treatments. Platelets play a vital role in oncology, hematology, traumatology, and general surgery (5, 6). Oncological and hematological treatments often require the use of fresh platelets, whereas patients undergoing trauma and who are undergoing general surgical procedures typically have no specific age preference (2, 7). As platelets approach their expiration date, their efficiency decreases, and the risk of bacterial contamination increases (8). Ignoring platelet age during transfusions can create compatibility issues and adverse transfusion reactions (7, 9–12). An article (13) discussed the demand for perishable products due to age discrimination. When specific blood types and platelet ages are unavailable, shortages may occur. The use of compatible blood units rather than exact matches (2, 14). Platelet transfusions are ensured to be compatible through cross-matching, minimizing immune reactions, reducing transfusion-transmitted infections, and enhancing transfusion efficacy (4, 15).

Platelets are collected from human donors using two primary methods: collection of apheresis platelets, also known as single donor platelets (SDPs), and collection of the platelet concentrate, also known as random donor platelets (RDPs) (16). The presence of the Rh factor can vary between SDPs and RDPs (17–19). Failure to consider the Rh factor during platelet transfusions may lead to alloimmunization and, in rare cases, hemolysis (18, 20, 21). To mitigate the risk of alloimmunization for Rh-negative patients, SDP is preferable when compatible blood groups are available. For exact matches, either the SDP or RDP can be utilized (17). Therefore, the Rh factor is crucial in platelet transfusions to ensure compatibility and minimize the risk of adverse reactions. Blood subtypes are often neglected in platelet transfusions because of limited awareness or shortages of specific blood types. In 1939, Ikin et al. (22) estimated the frequencies of $A_{1}$ and $A_{2}$ blood types in the population of England for $A_{1}A_{2}BO$ system. Several researchers have been discussed the importance of subtypes of A in blood transfusion practices (22–29). Despite advancements in the ABO blood group system, a significant gap remains in the comprehensive classification of the $A_{1}A_{2}BO$ system. Prioritizing the most in-demand blood groups during the matching process is essential, as this can substantially reduce the risk of transfusion reactions (30). In terms of subtypes, $A_{1}$ and $A_{2}$ are categorized as type A, whereas $A_{1}B$ and $A_{2}B$ are classified as type AB.

**Figure 1:**
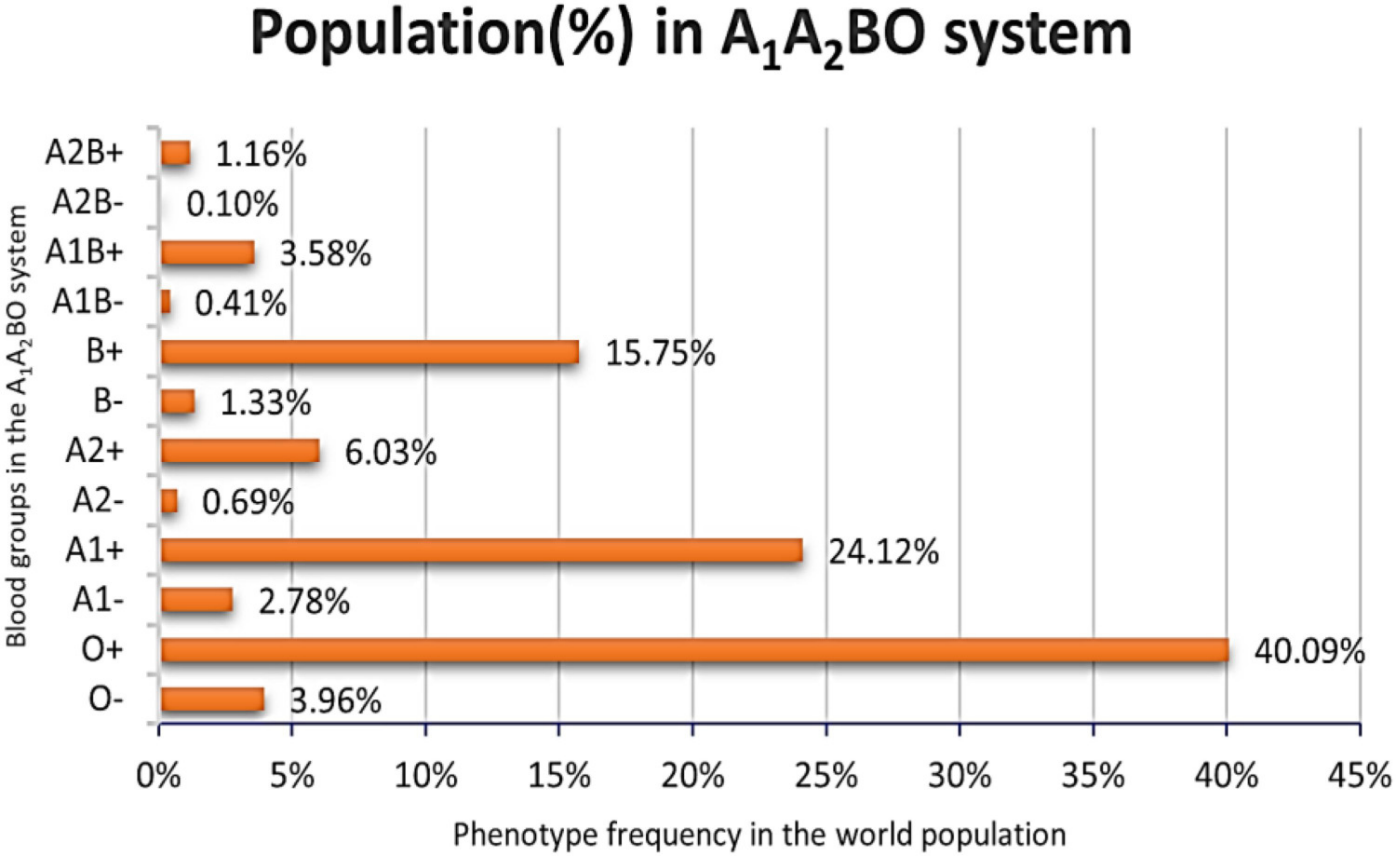
World population percentage of the $A_{1}A_{2}BO$ system.

**Figure 2:**
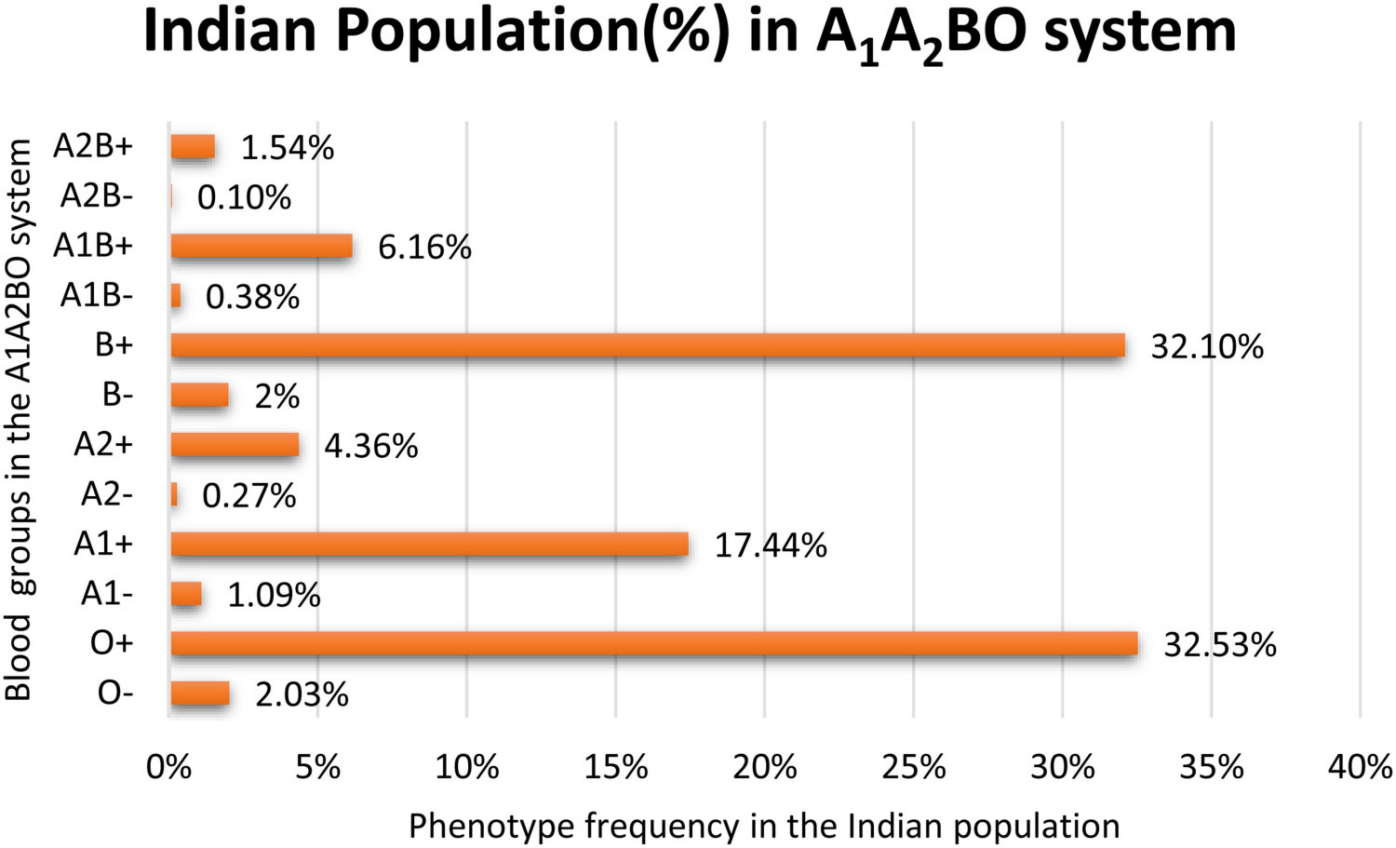
Indian population percentage of the $A_{1}A_{2}BO$ system.

**Figure 3:**
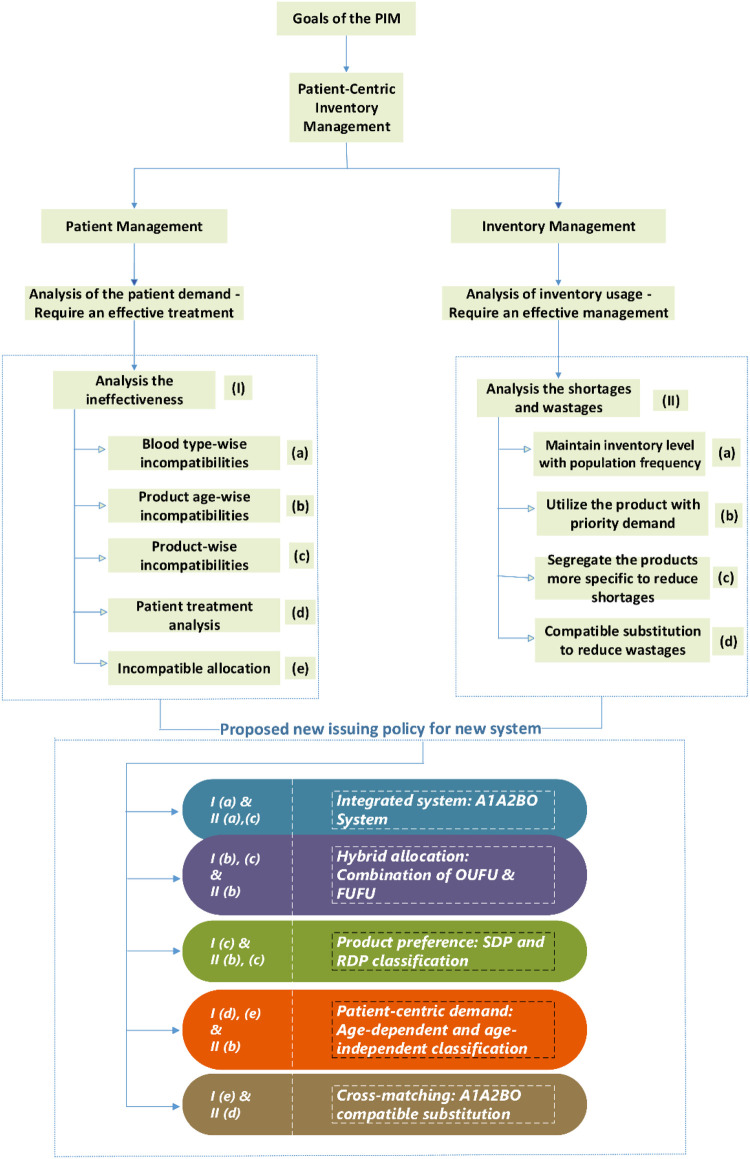
Architecture of the heuristic model illustrating a framework for patient-centric blood bank management.

According to several literature and medical articles (22–26, 31–33), approximately 80% of individuals with blood type A belong to the $A_{1}$ subtype, while 20% belong to the $A_{2}$ subtype. The population percentages for the $A_{1}A_{2}BO$ system are derived using the world population dataset for the ABO system, as illustrated in Figure 1 (34). In accordance with (32), Figure 2 depicts the frequency of the $A_{1}A_{2}BO$ phenotypes in the Indian population. Figures 1, 2 show that the phenotype frequencies of the individuals with blood type $A_{2}+$ are greater than those with $A_{1}B+$, *B−*, and $A_{1}B-$. Integrating blood subtypes into the ABO system can help address shortages in specific blood types and improve service levels. Although the percentage may appear small, India's position as the second-most populous country underscores the need for system upgrades to achieve sustainable health goals. However, current inventory management systems often prioritize inventories for lower-frequency phenotypes. To enhance healthcare treatment outcomes while considering both patient and population perspectives, this research article proposes an optimized PIM-issuing policy tailored to the $A_{1}A_{2}BO$ system (refer to Figure 3).

The widely recognized blood subgroups $A_{1}$ and $A_{2}$ are pivotal in the design of PIM-issuing policies (25–27). The necessity of categorizing platelets into $A_{1}$ and $A_{2}$ subgroups is supported by evidence that $A_{2}$ individuals exhibit minimal expression of A antigens on thrombocytes (35, 36). Incorporating the $A_{1}A_{2}BO$ system into the PIM framework enhances operational performance and allocation. Integrating the $A_{1}A_{2}BO$ system into the RBC compatibility table alongside an age-dependent cross-matching policy has been identified as an effective strategy to increase safety standards (28, 37). In platelet transfusion, O+ is universally considered a recipient in the ABO system and is never a donor. The same principle applies to the $A_{1}A_{2}BO$ system. From (1), the platelet compatibility preferences are provided in Table 1, where O+ serves as the universal recipient in platelet transfusions. Requests for blood types $A_{2}$ and $A_{2}B$ are typically associated with blood types A and AB in the ABO system, which could increase the risk of incompatible reactions, as mentioned in Table 2 (21). These reactions can be reduced with the implementation of the $A_{1}A_{2}BO$ blood group system. Subtype-aware allocation reduces based on broad-compatibility substitutions and enhances precise allocation. For routine transfusion, exact matches are preferred; compatible substitutions are considered only under constrained inventory conditions. The inclusion of the $A_{1}$ and $A_{2}$ subgroups provide a more precise representation of transfusion practices than traditional ABO-based models. Blood banks and hospitals are implementing the OUFU policy when blood products are distributed within their inventory management (42–44). The storage duration of blood products has been shown to affect patient outcomes, with evidence indicating that transfusing fresher units may confer modest but clinically relevant benefits (24, 25, 40, 41) (see Table 2). It is hypothesized that providing fresh platelets, particularly to children, pregnant individuals, cancer patients, and patients in critical emergencies, can substantially improve operational performance.

**Table 1:** $A_{1}A_{2}BO$ platelet compatibility between donor and recipient types.

Donor	Recipient											
	O−	O+	$A_{1}-$	$A_{1}+$	$A_{2}-$	$A_{2}+$	B−	B+	$A_{1}B-$	$A_{1}B+$	$A_{2}B-$	$A_{2}B+$
O−	*	*										
O+		*										
$A_{1}-$	*	*	*	*								
$A_{1}+$		*		*								
$A_{2}-$	*	*			*	*						
$A_{2}+$		*				*						
B−	*	*					*	*				
B+		*						*				
$A_{1}B-$	*	*	*	*			*	*	*	*		
$A_{1}B+$		*		*				*		*		
$A_{2}B-$	*	*			*	*	*	*			*	*
$A_{2}B+$		*				*		*				*

*, compatibility; blank, incompatibility.

**Table 2:** Summary of the integration of peer-reviewed medical data and clinical expert inputs for model formulation.

Medical research articles/Case study	Reactions	Drawbacks	Mitigation strategy in PIM
(1, 16, 17, 19, 21, 38)	Risk of RhD alloimmunization	Ignorance of the Rh factors	SDP and RDP Product segregation
(24, 25, 39)	Allergic and febrile reactions	Ignorance of the subtypes	Classification of $A_{1}\;$And non-$A_{1}$
(1, 18, 23, 24, 31)	Minor ABO incompatibility	Phenotype frequency Analysis of $A_{1}A_{2}BO$	Integration of the ABO system into $A_{1}A_{2}BO$ system
(24, 25, 40, 41)	Lower CCI increments	Ignorance of the age of the products	Demand was analyzed, and the issuing process was formulated using a combination of FUFU and OUFU, with age-mismatch preferences prioritizing demand.
(1, 15, 18, 21, 41)	Risk of hemolysis	Cross-matching; Incompatibilities and Product classification due to the minimal amount of RBC in platelets	$A_{1}A_{2}BO$ compatibility substitution table and the product classification

The proposed policy prioritizes product allocation based on the following hierarchy:

- $A_{1}A_{2}BO$ group compatibility
- Rh factor compatibility and
- Product age, whenever feasible (refer to Tables 1, 3, 4)

**Table 3:** Product-preference table.

Products	Demand by	
	Rh−ve	Rh+ve
SDP	I	II
RDP	II	I

**Table 4:** Age-mismatch preference table for an exact match.

Shelf-life	Demand by	
	Rh−ve	Rh+ve
	Fresh	Ordinary
Fresh	I (FUFU)	II (OUFU)
Ordinary	II (FUFU)	I (OUFU)

This patient-centric approach is designed to improve the alignment between platelet allocation decisions and patient-specific demand characteristics within the inventory system. “Patient-centric” refers to an allocation that prioritizes compatibility constraints, demand characteristics, and product-specific requirements to enhance the match available inventory with patient needs. The proposed model focuses on improving operational decision-making in platelet issuing policies rather than directly evaluating patient-level clinical outcomes. Research on platelet transfusions should focus on determining optimal doses for different patient populations (45). The Association for the Advancement of Blood and Biotherapies (AABB) emphasizes the importance of platelet transfusions to individual patient needs, thereby enhancing the overall quality of care and service (3, 46, 47). This patient-centric approach not only prioritizes the well-being of those we serve but also elevates the standards of our health services. This article introduces an enhanced PIM issuing policy that integrates a cross-matching model fortified by the incorporation of the $A_{1}A_{2}BO$ system to address patient-centric demands. To address the challenges in managing platelet inventories and transfusions, this article proposes a hybrid issuing policy tailored to the $A_{1}A_{2}BO$ system. Age-differentiated demand and product preference for the Rh factor, to support patient-centric allocation (refer to Figure 3). The overarching goal of the healthcare sector remains to minimize waste and shortages. Accordingly, the proposed PIM strategy prioritizes the allocation of appropriate platelet products to meet the demands of healthcare centers within the $A_{1}A_{2}BO$ system while effectively reducing waste and shortages (4, 14, 44, 48). There are fewer articles on the issuing policy dealing with Arena simulations. The ARENA simulation environment is primarily for queuing analysis. In this article, product allocations from management are used in inventory management. The term “heuristic,” as used in this article, denotes a structured, rule-based issuing policy. This policy is developed based on clinical compatibility and operational constraints, rather than on a metaheuristic optimization approach.

This research highlights the theoretical work and policy formulations that underpin clinical hypotheses in blood banking and transfusion medicine, as shown in Table 2. Upgrading blood bank systems has been identified as a pivotal step toward validating and reinforcing these hypotheses, a need underscored during the pandemic (49). To support this effort, a research questionnaire aligned with the proposed policies was developed and reviewed by clinical experts. Their feedback provided alternative methods to validate the issuing policy in the upgraded ABO system, offering valuable insights into its feasibility and practical implementation (see the Supplementary Material).

This study focused on developing a simulation-based operational framework to evaluate heuristic platelet issuance policies. The proposed model is intended to support decision-making by analyzing inventory performance under varying demand and compatibility conditions, rather than directly assessing clinical outcomes. To enhance the understanding of the proposed framework, numerical examples and scenario-based simulations are provided to illustrate the behavior of the system under different operational scenarios.

## 2 Literature review

Platelet transfusions based on operational performance are vital to meet the transfusion threshold (50). The evidence-based guidelines directly affect inventory management decisions by establishing clear criteria for platelet issuance, potentially reducing unnecessary transfusions and optimizing limited resources. Human factors influencing inventory management are often overlooked in existing models (51).

### 2.1 Research gap

This proposed work address a critical gap in the literature (refer to Table 2) exploring the practical application of blood subtype classification within groups in an area of significant academic interest that has yet to be fully integrated into real-world blood bank operations. Since 1940, research on subtype classification has established phenotype frequencies for the $A_{1}A_{2}BO$ blood group system (22). However, current blood bank systems continue to depend primarily on the ABO and Rh factor systems, and often overlook these crucial subtypes. No prior studies have addressed the $A_{1}A_{2}BO$ system classification represented in blood banking. From a medical perspective, the model introduces an innovative issuance policy to meet practical needs in blood inventory management (see Figure 3). Platelets are not a single product stored in blood banks. As discussed earlier, platelet components are operationally classified into SDP and RDP, with product selection influenced by treatment type, blood group, and Rh compatibility. However, most existing platelet inventory management studies (2, 4, 7, 9, 10, 43, 48) consider platelets as a single product and overlook this critical classification. Blood banks manage SDP and RDP separately. Hence, observe product differentiation to establish a more accurate, patient-centric platelet allocation framework. The Rh factor considerations, ensuring that Rh-negative patients receive SDP exclusively during crossmatching. Moreover this issuing policy and a compatible substitute to mitigate platelet shortages. A key contribution of this study is its differentiation between SDP and RDP in inventory management, a distinction that has been unnoticed in the PIM. Current platelet allocation practices lack a formalized compatibility hierarchy, limiting systematic evaluation and optimization of issuing policies. These gaps, represents a significant step toward optimizing platelet inventory management practices and advancing operational performance.

### 2.2 Problem identification

The research model is structured to achieve the following objectives:

- *Minimize platelet wastage:* Due to their limited lifespan (5–7 days), platelets are more prone to wastage than other blood products are, significantly affecting the efficiency of blood bank inventories.
- *Account for ABO antigens and RBC content:* Platelets naturally carry ABO antigens on their surfaces, but the amount of residual RBCs varies between platelet types. Specifically, each RDP contains approximately 0.036 mL of RBCs, while each SDP contains just 0.00043 mL (52).
- *Develop compatibility preference tables:* Establishing an optimal compatibility preference table that incorporates blood group, product age, and platelet type is vital to minimizing ABO incompatibility and RhD alloimmunization.
- *Promote patient-centric distribution strategies:* Despite the growing emphasis on patient-centric approaches in healthcare (53), traditional blood bank practices predominantly follow OUFU policy, which restricts the potential for personalized care.
- *Utilize blood subtypes effectively:* While individuals with the $A_{2}$ subtype can donate platelets to both O and B blood groups, current blood bank practices have not yet incorporated the use of these subtypes (24, 54).
- *Mitigate ABO incompatibility:* Medical literature confirms that ABO incompatibility reduces the corrected count increment (CCI). As a result, standard protocols recommend using ABO-compatible platelets to improve allocation effects.
- *Address operational risks associated with high-titer O-group donors:* Utilizing SDP from such donors poses significant risks to operational performance and requires vigilant oversight and robust policy frameworks.
- *Optimize platelet allocation for specific patient needs:* In cancer patients, young platelets (1–2 days old) significantly improve service levels (2, 7, 9). In contrast, platelets of any age are acceptable for trauma (55) and general surgical procedures (2, 4, 56). This flexibility supports the use of a hybrid allocation approach that combines FUFU and OUFU methods.

### 2.3 Problem statement

In transfusion medicine, PIM plays a critical role in balancing two essential goals: optimizing patient care and ensuring efficient inventory utilization. Despite advancements in storage and compatibility testing, current platelet issuing policies face significant limitations that hinder both allocation and operational efficiency.

From the patient care perspective, existing protocols often fail to notice the important clinical variables such as ABO subtyping $\left(A_{1}\;\mathrm{and}\;A_{2}\right)$, age, type of platelet products (SDP vs. RDP), and patient-specific treatment. These inappropriate product allocation, transfusion inefficacies, and an increased risk of alloimmunization, particularly for patients with rare blood phenotypes or recurrent transfusion needs. From the IM perspective, blood banks face recurring challenges, including mismatches between supply and population-specific demand, suboptimal utilization of time-sensitive platelet units, high wastage due to expiration, and the absence of structured substitution strategies to mitigate shortages. The lack of integration between clinical demand characteristics and logistics for issuing exacerbates these inefficiencies. These dual challenges underscore the need for a robust, data-driven, and patient-centric issuing framework. There is a clear gap in the literature and in practice for a policy that integrates compatibility-aware allocation, age-prioritized issuance, and product-specific differentiation, while remaining adaptable across various healthcare settings with differing technological and operational capacities. To enhance clarity, the workflow and model parameters are illustrated systematically in Figure 3.

### 2.4 Limitations

The limitations of the proposed model are outlined below.

- The implementation of age-independent and age-dependent demand is suitable for regional blood banks with high platelet transactions and may require adaptation for smaller or low-demand settings.
- The model incorporates the $A_{2}$ subtype classification to improve compatibility handling within the allocation. However, this approach expects system-level assumptions and does not directly assess clinical transfusion outcomes.
- The demand classification us based on expert suggestions and the designed assumptions: ordinary and emergency. The model supports structured decision-making, practical implementation may vary depending on institutional protocols and operational constraints.
- The performance of the proposed model is influenced by the phenotype frequency. The model is designed to improve service levels, its applicability may vary across regions with different demographic characteristics. Further integration with advanced technologies such as IIoT may further enhance implementation, whereas in resource-constrained settings, adoption may depend on the availability of trained personnel.
- This study is based on retrospective, anonymized blood bank data and simulation-based analysis, including a 15-day (three ordering cycles); the findings should be interpreted as operational performance within a simulation environment rather than clinically validated outcomes. Further clinical validation using real-world data is needed to assess practical applicability.
- The proposed model considers both compatibility requirements and overall inventory conditions; Yet its implementation requires careful consideration to ensure balanced utilization across all blood groups and to avoid unintended imbalance in product distribution.
- Although $A_{1}/A_{2}$ subtyping has limited clinical impact in routine platelet transfusion practices, it is incorporated into this study to examine whether phenotype classification can improve allocation and inventory efficiency.

## 3 Materials and methods

This issuing policy for the PIM system is structured into four key subsections: clinical validation and expert input, model description, schematic diagram, and mathematical formulation.

### 3.1 Clinical context and expert input

To integrate clinical context into the proposed PIM-issuing policy model, a structured questionnaire was developed from the literature and subsequently used to gather expert input. This questionnaire was distributed to three healthcare professionals: (i) a surgeon with extensive experience in transfusion-related decision-making, (ii) a technical supervisor from a blood bank responsible for managing platelet inventories, and (iii) the director of a government transfusion center with oversight of patient care manager and operational protocol. These experts were selected for their direct involvement in transfusion medicine and blood bank operations. The questionnaire addressed key clinical considerations, such patient classification during emergencies, optimal shelf life for platelet transfusions, substitution allowances, and subtype classification to control transfusion reactions (see Table 2). The responses were used to obtain preliminary qualitative insights into the relevance and applicability of the model assumptions.

It is important to note that this expert input is limited in scope, as it is based on a small number of participants and does not follow a formal validation methodology. Therefore, it is treated as supportive qualitative feedback rather than clinical validation. No quantitative statistical analysis was performed on expert responses.

For transparency and reproducibility, we have provided the complete questionnaire and the corresponding expert responses in the Supplementary Material.

### 3.2 Model description

**Figure 4:**
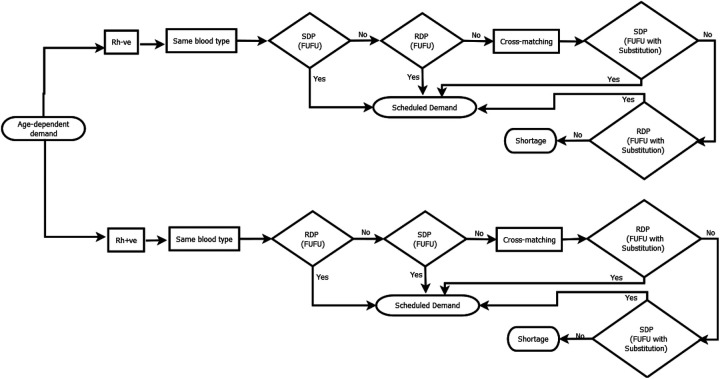
Age-dependent demand issuing policy (cases 1 and 2).

**Figure 5:**
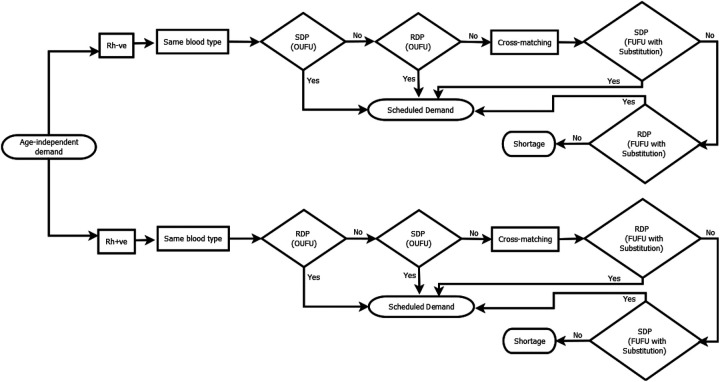
Age-independent demand issuing policy (cases 3 and 4).

Platelet demand can be age-dependent or age-independent, depending on the surgeon's request or the patient's treatment, such as emergency care or general surgery. Platelet bags undergo processing and testing for 1–2 days, and they become available in inventory after 5–7 days. As such, this model assumes a platelet shelf life of 5 days. Ordinary blood (with a remaining shelf life of 3–1 days) is limited to satisfying demand for its corresponding blood group, whereas fresh blood (with a remaining shelf life of 5–4 days) can meet demand for both groups. Products are issued on the basis of the Rh factor and treatment requirements (refer to Tables 3, 4). Medical experts also consider the patient's age, sex, and complications before performing the transfusion. The inventory system uses the FUFU policy for age-dependent demand and the OUFU policy for age-independent demand. In cases of insufficient platelet age matching, an age mismatch is permitted, followed by $A_{1}A_{2}BO$ compatible substitution, following the FUFU policy. Before considering $A_{1}A_{2}BO$ substitution, the Rh factor is prioritized. RDP or pooled platelets are preferred for Rh-positive patients, whereas SDP is the first choice for Rh-negative patients. The FUFU policy is always adhered to during cross-matching (refer to Table 4). The $A_{1}A_{2}BO$ classification is included in the cross-matching process to minimize compatibility risks, with AB blood type donors being the highest-priority donors. The proposed design aims to improve platelet availability and management, reduce waste, and enhance operational performance. A graphical overview of the model is provided for clarity, with a detailed schematic representation illustrating the process (see Figures 4, 5). On the basis of the above discussion, the platelet demand for patient treatment and the Rh factor can be classified as follows:

1. Age-dependent and Rh-negative
2. Age-dependent and Rh-positive
3. Age-independent and Rh-negative
4. Age-independent and Rh-positive

### 3.3 Schematic representation of the issuing policy

The heuristic platelet issuing policies for different demand types are classified and shown in Figures 4, 5.

### 3.4 Mathematical formulation

A description of the notations used in this mathematical model is provided in Table 5.

**Table 5:** List of notations used in the mathematical model.

Notation	Description
t	The time period t=1,2,…,T is the finite time horizon
α	The age of blood; α=1,2,…,M, where *M* is the maximum shelf life of 5 days
k	k={1,2,…,12} = the roster of blood types $\{O-,\;O+,\;A_{1}-,\;A_{1}+,\;A_{2}-,\;A_{2}+,B-,B+,A_{1}B-,A_{1}B+,\;A_{2}B-,A_{2}B+$
$X^{t}$	The platelet inventory status of twelve blood types at *t* for SDPs
$Y^{t}$	The platelet inventory status of twelve blood types at *t* for RDPs
$Z^{t}$	The platelet inventory status of twelve blood types at *t* in both SDP and RDP
$V_{k}^{d}$	The vector indicating the order-up level for blood type *k* in day *d*, where d=1,2,…,5
$SX_{k}^{t}$	The total availability of a specific blood type *k* for SDPs at period t
$RY_{k}^{t}$	The total availability of a specific blood type *k* for RDPs at period t
$BL_{k}^{t}$	The backlog of *k* for the age-independent demand is not satisfied with the exact match in t
$BL_{k}^{{}^{\prime }t}$	The backlog of *k* for the age-dependent demand is not satisfied with the exact match in t
$d_{k}^{t}$	The age-independent demand of *k* in t
$d_{k}^{{}^{\prime }t}$	The age-dependent demand of *k* in t
$k_{+}$	Rh + for the blood group k
$k_{-}$	Rh- for the blood group k
$SCM_{k}^{t}$	SDP cross-matched platelets in SDPs of *k* at the end of period t
$RCM_{k}^{t}$	RDP cross-matched platelets in RDPs of *k* at the end of period t
$SO_{k}^{t}$	Wastage of the platelets in SDPs of *k* at the end of period t
$RO_{k}^{t}$	Wastage of the platelets in RDPs of *k* at the end of period t
$CS_{k}$	Complete shortage of *k* throughout the planning period T
$CO_{k}$	Complete obsolescence of the blood group *k* throughout the planning period T

Let the matrices $X^{T}$ and $Y^{T}$ depict the inventory status of platelets across the twelve blood group types *t* in SDP and RDP, respectively.

$$X^{t}=\begin{array}{c} O-\\ O+\\ \vdots \\ A_{2}B+ \end{array}\left[\begin{array}{cccc} x_{1,1}^{t} & x_{1,2}^{t} & \cdots & x_{1,M}^{t}\\ x_{2,1}^{t} & x_{2,2}^{t} & \cdots & x_{2,M}^{t}\\ \vdots & \vdots & \ddots & \vdots \\ x_{12,1}^{t} & x_{12,2}^{t} & \ldots & x_{12,M}^{t} \end{array}\right]$$ (1)

and

$$Y^{t}=\begin{array}{c} O-\\ O+\\ \vdots \\ A_{2}B+ \end{array}\left[\begin{array}{cccc} y_{1,1}^{t} & y_{1,2}^{t} & \cdots & y_{1,M}^{t}\\ y_{2,1}^{t} & y_{2,2}^{t} & \cdots & y_{2,M}^{t}\\ \vdots & \vdots & \ddots & \vdots \\ y_{12,1}^{t} & y_{12,2}^{t} & \ldots & y_{12,M}^{t} \end{array}\right]$$ (2)

Now the matrix $Z^{t}$ symbolizes the collective platelet inventory status of the $A_{1}A_{2}BO$ system at *t*,

$$Z^{t}=X^{t}+Y^{t},$$

where $z_{k,\alpha }^{t}=x_{k,\alpha }^{t}+y_{k,\alpha }^{t}\;$from Equations 1 and 2

$V_{k}^{d}$ specifies the vector representing the “order-up-to-level” for the blood type *k* in *d*, where d∈[1,2,…,5] and the overview of the decision matrix is as follows:

$$V=\begin{array}{c} O-\\ \vdots \\ A_{2}B+ \end{array}\;\left[\begin{array}{ccc} V_{1}^{1} & \cdots & V_{1}^{5}\\ \vdots & \ddots & \vdots \\ V_{12}^{1} & \cdots & V_{12}^{5} \end{array}\right]\;$$ (3)

The vector form of the available units of all twelve blood types across all age categories is as follows:

$$\left[\begin{array}{ccc} \overset{M}{\underset{a=1}{\sum }}z_{1,a} & \overset{M}{\underset{a=1}{\sum }}z_{2,a} & \begin{array}{cc} \ldots & \overset{M}{\underset{a=1}{\sum }}z_{12,a} \end{array} \end{array}\right]$$ (4)

The inventory of all age categories of a specific blood group *k* is indicated as follows:

$$Z_{k}^{t}=\left[\begin{array}{cccc} z_{k,1}^{t} & z_{k,2}^{t} & \ldots & z_{k,M}^{t} \end{array}\right]\;$$ (5)

where 1≤M≤6

The requested demand, categorized by age, for a blood type *k* is represented as follows:

$$\rho_{k}=\{\begin{array}{ccc} 1 & : & \mathrm{if}\;\mathrm{the}\;\mathrm{demand}\;\mathrm{of}\;\mathrm{the}\;\mathrm{type}\;k\;\mathrm{is}\;\mathrm{age}\;\mathrm{dependent}\\ 0 & : & \;\mathrm{if}\;\mathrm{the}\;\;\mathrm{demand}\;\mathrm{of}\;\mathrm{the}\;\mathrm{type}\;k\;\mathrm{is}\;\mathrm{age}\;\mathrm{independent} \end{array}\;\;\;$$ (6)

The vector representation of demand priority for each blood type is provided as

$$\rho =\left[\begin{array}{cccc} \rho_{1} & \rho_{2} & \ldots & \rho_{k} \end{array}\right]\;\mathrm{for}\;k=1,2,\ldots ,12$$

The age of the platelets can be classified as,

$$\alpha =\{\begin{array}{ccc} 1\leq \alpha \leq 2 & : & \mathrm{if}\;\mathrm{young}\;\mathrm{platelets}\;\\ 3\leq \alpha \leq M & : & \;\mathrm{if}\;\mathrm{ordinary}\;\mathrm{platelets} \end{array}\;$$ (7)

where α is a discrete variable.

The availability of young platelets within a specific blood group *k* in SDPs at period *t* refers to platelet units of the same age or exceeding the age Φ in an exact match as follows:

$$\;SX_{k}^{t}(\Phi )=\overset{2}{\underset{\alpha =1}{\sum }}x_{k,\alpha }^{t}\;$$ (8)

Similarly, the availability of young platelets in a specific blood group k in RDP at period t is indicated by platelet units of the same age or exceeding the age of Φ in an exact match as follow:

$$\;RY_{k}^{t}(\Phi )=\overset{2}{\underset{\alpha =1}{\sum }}y_{k,\alpha }^{t}$$ (9)

Let $SX_{k}^{t}(\Psi )$ represent the ordinary platelet units of the same blood type *k* at period t in SDPs that are “equal to or younger than Ψ ”, where

$$\;SX_{k}^{t}(\Psi )=\overset{M}{\underset{\alpha =3}{\sum }}x_{k,\alpha }^{t}\;$$ (10)

Let $RY_{k}^{t}(\Psi )$ represent the ordinary platelet units of the same blood type *k* at period t in RDPs that are “equal to or younger than Ψ ” as follows:

$$RY_{k}^{t}(\Psi )=\overset{M}{\underset{\alpha =3}{\sum }}y_{k,\alpha }^{t}$$ (11)

Owing to treatment and the Rh factor, alterations in order quantities and substitution procedures have led to daily fluctuations in inventory status. The following scenarios are examined on the basis of demand priority and the Rh factor (refer to Figures 4, 5). Equations 1 to 11 represents the inventory level to demand arrival processes in the PIM.

#### 3.4.1 Without substitution

Case 1: The demand is age-dependent $\left(\rho_{k}=1\right)$ for $k_{-}$

*Iteration 1*: Initially, demand is met by a perfect match with young platelets (FUFU).

Now, the backlog of $k_{-}$ is as follows:

$$\;\;\;\;BL_{k_{-}}^{\prime t}=[d_{k_{-}}^{\prime t}-SX_{k_{-}}^{t}(\Phi ){]}^{+}\;;\;t=1,2,\ldots ,T\;$$ (12)

where $[*{]}^{+}$ max(*,0)

*Iteration 2*:

If $BL_{k-}^{\prime t}\;>\;0$, then, the demand is fulfilled with young platelets of $k_{-}$ from RDP (FUFU).

Now, the backlog of $k_{-}$ is,

$$\;\;\;\;BL_{k_{-}}^{\prime t}=[d_{k_{-}}^{\prime t}-RY_{k_{-}}^{t}(\Phi ){]}^{+}\;;\;t=1,2,\ldots ,T$$ (13)

Case 2: The demand is age-dependent for $k_{+}$

*Iteration 1*:

Initially, the demand is met by its exact match with young platelets (FUFU).

Now, the backlog of $k_{+}$ is,

$$BL_{k_{+}}^{\prime t}=[d_{k_{+}}^{\prime t}-RY_{k_{+}}^{t}(\Phi ){]}^{+}\;;\;t=1,2,\ldots ,T$$ (14)

*Iteration 2*:

If $BL_{k_{+}}^{\prime t}\;>\;0$, then the demand is fulfilled with young platelets of $k_{+}$ from SDP (FUFU).

Now, the backlog of $k_{+}$ is,

$$\;\;\;\;BL_{k_{+}}^{\prime t}=[d_{k_{+}}^{\prime t}-SX_{k_{+}}^{t}(\Phi ){]}^{+}\;;\;t=1,2,\ldots ,T$$ (15)

Case 3: The demand is age-independent $\left(\rho_{k}\;=0\right)$ for $k_{-}$

*Iteration 1*: Initially, it is fulfilled by an exact match with ordinary platelets (OUFU).

Now, the backlog of $k_{-}\;$ is,

$$BL_{k_{-}}^{t}=[d_{k_{-}}^{t}-SX_{k_{-}}^{t}(\Psi ){]}^{+}\;;\;t=1,2,\ldots ,T$$ (16)

*Iteration 2*: If $BL_{k_{-}}^{t}\;>\;0$, then, the demand is fulfilled by an exact match with ordinary platelets (OUFU).

Now the backlog of $k_{-}$ is,

$$BL_{k_{-}}^{t}=[d_{k_{-}}^{t}-RY_{k_{-}}^{t}(\Psi ){]}^{+}\;;\;t=1,2,\ldots ,T$$ (17)

Case 4: The demand is age-independent of blood type $k_{+}$

*Iteration 1*: Initially, demand is fulfilled by exact matches with ordinary platelets (OUFU).

Now, the backlog of $k_{+}$ is,

$$\;BL_{k_{+}}^{t}=[d_{k_{+}}^{t}-RY_{k_{+}}^{t}(\Psi ){]}^{+}\;;\;t=1,2,\ldots ,T$$ (18)

*Iteration 2*: If $BL_{k_{+}}^{t}\;>\;0$, then the demand can be fulfilled by an exact match with ordinary platelets (OUFU).

Now, the backlog of $k_{+}$ is,

$$BL_{k_{+}}^{t}=[d_{k_{+}}^{t}-SX_{k_{+}}^{t}(\Psi ){]}^{+}\;;\;t=1,2,\ldots ,T$$ (19)

Equations 12 to 19 represents the heuristic processes for without substitution allocation rules.

#### 3.4.2 With substitution

##### 3.4.2.1 For cases 1 & 3: (for Rh−)

Suppose that the age-dependent and age-independent demands for $k_{-}$ persist, denoted as $BL_{k_{-}}^{\prime t}$ and $BL_{k_{-}}^{t}>\;0$, respectively; then the initial demand can be addressed through substitution or a series of substitutes, adhering to the FUFU policy for SDP as per the compatibility table (refer to Table 4). If the backlog remains, then the substitution is followed by the FUFU policy for RDPs.

In SDP and RDP, the total blood unit utilized to fulfill $k_{-}$ solely through substitution is represented by $SCM_{k}^{t}$. Based on the preceding discussion, the observed shortages, both age-dependent and age-independent, of $k_{-}$ after period *t* are as follows:

$$S_{k_{-}}^{\prime t}=[BL_{k_{-}}^{\prime t}-SCM_{k}^{t}-RCM_{k}^{t}{]}^{+}\;;\;t=1,2,\ldots T$$ (20)

and

$$S_{k_{-}}^{t}=[BL_{k_{-}}^{t}-SCM_{k}^{t}-RCM_{k}^{t}{]}^{+}\;;\;t=1,2,\ldots ,T$$ (21)

respectively.

##### 3.4.2.2 For cases 2 & 4: (for Rh+)

Suppose that the age-dependent demand and the age-independent demand for $k_{+}$ are still $BL_{k_{+}}^{\prime t}$ and $BL_{k_{+}}^{t}>0$, respectively. In such scenarios, the initial demand can be met through substitution, or a series of substitutes must be utilized following the FUFU policy for RDPs as per the compatibility table (refer to Table 4). If there is still a backlog, the FUFU policy applies to SDPs.

For SDPs and RDPs, a comprehensive blood unit is used to meet the blood group requirements $k_{+}$ exclusively through substitution is denoted as $RCM_{k}^{t}$. As discussed above, the observed age-dependent and age-independent scarcities of blood groups $k_{+}$ after period *t* are as follows:

$$S_{k_{+}}^{\prime t}=[BL_{k_{+}}^{\prime t}-SCM_{k}^{t}-RCM_{k}^{t}{]}^{+}\;;\;t=1,2,\ldots ,T$$ (22)

and

$$S_{k_{+}}^{t}=[BL_{k_{+}}^{t}-SCM_{k}^{t}-RCM_{k}^{t}{]}^{+}\;;\;t=1,2,\ldots ,T\;$$ (23)

respectively. Equations 20 to 23 represents the shortages after processed the heuristic procedure with substitution.

Hence, from Equations 22 and 23, the shortage of the period *t* is as follows:

$$S_{k}^{t}=\overset{12}{\underset{k=1}{\sum }}S_{k}^{\prime t}+\overset{12}{\underset{k=1}{\sum }}S_{k}^{t}$$ (24)

Following the issuance process outlined above, the updated inventory states $x_{k,\alpha }^{t}$ and $y_{k,\alpha }^{t}$ can be determined as follows:

$$x_{k,\alpha }^{t}\;=\left\{ \begin{array}{c} {\left[x_{k,\alpha }^{t}-d_{k}^{{}^{\prime }t}-d_{k}^{t}-SCM_{k}^{t}\right]}^{+}:\alpha =1\;\\ {\left[x_{k,\alpha }^{t}-[d_{k}^{t}-d_{k}^{{}^{\prime }t}-SX_{k}^{t}(\alpha +1)-SCM_{k}^{t}\right]}^{+}{]}^{+}:2\leq \alpha \leq M\;\\ {\left[x_{k,\alpha }^{t}-{\left[d_{k}^{t}-SCM_{k}^{t}\right]}^{+}\right]}^{+}:\alpha =M\; \end{array}\right.$$ (25)

$$y_{k,\alpha }^{t}\;=\{\begin{array}{ccc} {\left[y_{k,\alpha }^{t}-d_{k}^{{}^{\prime }t}-d_{k}^{t}-SCM_{k}^{t}\right]}^{+}\; & :\; & \alpha =1\;\\ {\left[y_{k,\alpha }^{t}-[d_{k}^{t}-d_{k}^{{}^{\prime }t}-RY_{k}^{t}\left(\alpha +1\right)-RCM_{k}^{t}\right]}^{+}{]}^{+}\; & :\; & 2\leq \alpha \leq M\;\\ {\left[y_{k,\alpha }^{t}-{\left[d_{k}^{t}-CM_{k}^{t}\right]}^{+}\right]}^{+}\; & :\; & \alpha =M\; \end{array}$$ (26)

As illustrated in Equations 25, 26, each blood unit is allocated to fulfill its corresponding demand, contingent on both its age and blood group. When the platelet age reaches *M*, they are either utilized within the week or become obsolete. Consequently, the platelet inventory does not contain any units aged older than *M*, $SX_{k}^{t}(m+n)=0$, and $RY_{k}^{t}(m+n)=0$, where n>0.

First, for a platelet age of α(2≤α≤M) all its platelet units are older than α and are allocated for age-independent $\left(d_{j}\right)$ and age-dependent demand $\left({d^{\prime }}_{j}\right)$ satisfied by the FUFU policy. Hence the terms $\left[d_{k}^{t}+d_{k}^{\prime t}-SX_{k}^{t}(\alpha +1)+SCM_{k}^{t}\right]$ and $\left[d_{k}^{t}+d_{k}^{{}^{\prime }t}-RY_{k}^{t}(\alpha +1)+RCM_{k}^{t}\right]$ refer to the remaining demand after substitution.

The expired units in SDPs at the end of period *T* are as follows:

$$SO_{k}^{t}=x_{k,M}^{t}\;;\;t=1,2,\ldots ,T$$ (27)

and the expired units in RDPs at the end of period *T* are as follows:

$$RO_{k}^{t}=y_{k,M}^{t}\;;\;t=1,2,\ldots ,T$$ (28)

Hence, from Equations 27 and 28, the overall wastage of *k* at the end of period *T* is,

$$O_{k}^{t}=SO_{k}^{t}+RO_{k}^{t}\;;\;t=1,2,\ldots ,T\mathrm{and}\;k=1,2,\ldots ,12$$ (29)

The inventory received new units, and all the platelet bags were aged 1 day. The following day, *t* + 1, commences with the current inventory as the starting point.

This is depicted for SDPs as follows:

$$x_{k,\alpha }^{t}\;=\{\begin{array}{ccc} \left[V_{k}^{\mathrm{MOD}(t,5)+1}-SX_{k}^{t}(\Psi )\right]\; & :\; & \alpha =1\;\\ x_{k,\alpha -1}^{t}\; & :\; & 1<\alpha \leq M\;\; \end{array}$$ (30)

where $V_{k}^{d}$ is the maximum inventory level of day *d*.

This is depicted for RDPs as follows:

$$y_{k,\alpha }^{t}\;=\{\begin{array}{ccc} \left[V_{k}^{\mathrm{MOD}(t,5)+1}-{\mathrm{RY}}_{k}^{t}(\Psi )\right]\; & :\; & \alpha =1\;\\ y_{k,\alpha -1}^{t}\; & :\; & 1<\alpha \leq M\; \end{array}$$ (31)

The modular division in the MOD(t,5)+1 function yields an index ranging from 1 to 5, representing 5 days.

Hence, from Equation 24, the complete shortage of *k* throughout the planning period *T* is,

$$CS_{k}=\overset{T}{\underset{t=1}{\sum }}S_{k}^{t};k=1,2,\ldots ,12\;$$ (32)

Similarly, from Equation 29, the complete outdatedness of *k* throughout the planning period *T* is,

$$CO_{k}=\overset{T}{\underset{t=1}{\sum }}O_{k}^{t};k=1,2,\ldots ,12\;$$ (33)

Hence, from Equations 32 and 33, the overall objective of the policy is to evaluate the reduction on a particular day *t* as follows in Equation 34:

$$min\overset{12}{\underset{k=1}{\sum }}CS_{k}+\overset{12}{\underset{k=1}{\sum }}CO_{k}$$ (34)

for *t*.

Based on this heuristic approach, a new operating protocol can be formulated for the PIM system.

The above objective is not explicitly optimized within the proposed framework, but is used as a performance measure to evaluate the effectiveness of the issuing policy. The rule-based allocation strategy aims to reduce shortage and obsolescence under different operational scenarios.

## 4 Results

### 4.1 Case studies

#### 4.1.1 Case study 1

In this study, the clinical case outlined in the article (1) is examined, and its implications for the proposed model to improve transfusion efficiency through inventory management (IM) are compared.

Patient medical details and SDP inventory availability are provided in Tables 6, 7, respectively.

**Table 6:** Details of the patient's clinical treatment and medical history.

Information	Patient details
Age of the patient	60 years
Gender	Female
Blood type	A negative
Disease	Newly diagnosed acute myeloid leukemia
Treatment	Chemotherapy-induced thrombocytopenia
Blood product request	Platelet

**Table 7:** Inventory details for SDP in the blood bank on a specific day.

Blood type	Age of the product
O+	5 days old (expiring that day at midnight)
B−	3 days old (expiring in 2 days)
A+	2 days old (expiring in 3 days)

Article (1) outlines the risks and benefits associated with each platelet option for the patient. Implementing the OUFU policy in IM prioritized options in the following order: O+, B−, and then A+ (refer to Table 7).

The patient's sex and age indicate no likelihood of future complications in hemolytic disease of the fetus and newborn (HDFN). The hospital's inventory management opts for O+ platelets. However, in consideration of the patient's age and sex, adhering to the FUFU policy is advised. Moreover, since the patient has blood type A but the specific blood subtype is unspecified, there is a possibility of an incompatible reaction post-transfusion or suboptimal performance in improving the CCI. A review of the data revealed that A+ SDP was associated with incompatible reactions, particularly RhD alloimmunization (57). Additionally, the RhD levels in SDP are lower than those in RDPs. Platelet transfusion aims to increase the patient's CCI. IM seeks to minimize both wastage and shortages while ensuring optimal service levels, and it is essential to consider compatibility preferences across the remaining blood types. This approach helps avoid wastage of the O blood type, which is the universal recipient in platelet transfusion, while maintaining adequate stock levels.

The decision path under the proposed model is as follows:

- Exact match (A−) → Not available
- Product substitution → Not required (SDP available)
- Compatibility check → A + and B− considered
- Priority rule (FUFU) → Selects the fresher unit (A+, 2 days old)

Thus, the model allocates an A + SDP based on compatibility and freshness criteria.

This allocation results in the following:

- Demand satisfied = 1
- Shortage = 0
- Wastage minimized by preserving near-expiry units (O+) for future use

#### 4.1.2 Case study 2

The incompatibilities and significance of the proposed system are derived from the medical literature and case studies on platelets (see Table 2). This analysis examines the frequency of phenotypes and the availability of the population. A specific case (27) highlighted an emergency involving blood type identification, as outlined in Table 2. Certain incompatibilities can lead to post-transfusion complications, including hemolytic and febrile reactions, acute lung injury, and alloimmunization. In obstetric emergencies, platelet transfusions (27) aim to increase the CCI post-transfusion. However, relying solely on this strategy without considering the factors in Table 2 may not increase the CCI.

The allocation process follows the hierarchical decision logic defined in the model:

- Exact match → Checked based on patient blood type
- If unavailable → Compatibility-based cross-matching is applied using the $A_{1}A_{2}BO$ system
- Substitution → Evaluated based on available inventory and product type
- Priority rule → FUFU or OUFU applied depending on demand characteristics

Using the phenotype frequency and demand structure, the model determines feasible allocation paths from the available inventory. The compatibility table (Table 2) is used to identify allowable substitutions, ensuring that clinically acceptable alternatives are selected.

The model output provides the following:

- Allocation decision based on the compatibility hierarchy
- Demand satisfaction status
- Impact on inventory levels

This case demonstrates that, even in the absence of exact matches, the proposed model enables systematic allocation through compatibility and substitution, reducing shortages while maintaining efficient inventory utilization.

In these cases, patients may not find an exact match within the current ABO system of IM. This challenge can be addressed through the following:

1. Integrating the ABO system into the $A_{1}A_{2}BO$ system,
2. Enabling clinicians to consult the $A_{1}A_{2}BO$ compatibility table to assess substitution availability and effectively utilize products from the inventory.

A numerical example is provided to analyze the model's flow, accounting for demand requirements and phenotype frequencies.

### 4.2 Numerical example

The platelet inventory is assessed daily, with a maximum shelf life of 5 days, and compatibility substitution is incorporated to ensure optimal usage.

Assume that the demand priority for age-dependent and no-age-preference platelets is established for day 1, with platelet orders placed up to the inventory level for this day for both SDP and RDP, as follows:

$$\rho =\left[\begin{array}{ccc} \begin{array}{ccc} \;\;1 & 1 & 0 \end{array} & \begin{array}{ccc} 1 & 0 & 0 \end{array} & \begin{array}{ccc} 0 & 1 & \begin{array}{ccc} 1 & 1 & \begin{array}{cc} 0 & 0\;\;\; \end{array} \end{array} \end{array} \end{array}\right]$$

$$U^{1}=\left[\begin{array}{ccc} \begin{array}{ccc} \;\;30 & 45 & 60 \end{array} & \begin{array}{ccc} 30 & 15 & 10 \end{array} & \begin{array}{ccc} 25 & 20 & \begin{array}{ccc} 20 & 35 & \begin{array}{cc} 8 & 5\;\;\; \end{array} \end{array} \end{array} \end{array}\right]$$

Let the initial SDP inventory for the 12 types on day 1 have a shelf life of 5 days.

$$X^{1}=\begin{array}{c} O-\\ O+\\ A_{1}-\\ A_{1}+\\ A_{2}-\\ A_{2}+\\ B-\\ B+\\ A_{1}B-\\ A_{1}B+\\ A_{2}B-\\ A_{2}B+ \end{array}\;\left[\begin{array}{ccccc} 7 & 1 & 2 & 0 & 0\\ 3 & 2 & 1 & 1 & 0\\ 6 & 2 & 2 & 0 & 2\\ 8 & 2 & 3 & 2 & 0\\ 1 & 1 & 0 & 0 & 0\\ 1 & 1 & 0 & 0 & 0\\ 3 & 7 & 0 & 1 & 2\\ 2 & 2 & 2 & 0 & 0\\ 4 & 2 & 3 & 1 & 0\\ 4 & 4 & 4 & 1 & 0\\ 3 & 1 & 0 & 0 & 0\\ 1 & 1 & 0 & 0 & 0 \end{array}\right]\;[\mathrm{by}\;\mathrm{Equation}\;1]$$

Let us begin with the RDP inventory status of the 12 blood types:

$$Y^{1}=\begin{array}{c} O-\\ O+\\ A_{1}-\\ A_{1}+\\ A_{2}-\\ A_{2}+\\ B-\\ B+\\ A_{1}B-\\ A_{1}B+\\ A_{2}B-\\ A_{2}B+ \end{array}\;\left[\begin{array}{ccccc} 5 & 4 & 1 & 0 & 0\\ 5 & 3 & 0 & 0 & 0\\ 10 & 1 & 2 & 0 & 0\\ 10 & 3 & 2 & 0 & 0\\ 0 & 0 & 0 & 0 & 0\\ 0 & 1 & 0 & 0 & 0\\ 0 & 8 & 3 & 1 & 0\\ 4 & 1 & 1 & 0 & 0\\ 4 & 1 & 0 & 0 & 0\\ 3 & 2 & 0 & 1 & 0\\ 0 & 1 & 0 & 0 & 0\\ 1 & 0 & 0 & 0 & 0 \end{array}\right]\;[\mathrm{by}\;\mathrm{Equation}\;2]$$

Hence, the available quantities of all types of platelets are as follows:

$$Z^{1}=\left[\begin{array}{ccc} \begin{array}{ccc} \;20 & 15 & 25 \end{array} & \begin{array}{ccc} 30 & 2 & 3\; \end{array} & \begin{array}{ccc} 25 & 12 & \begin{array}{ccc} 15 & 20 & \begin{array}{cc} 5 & 3\;\;\; \end{array} \end{array} \end{array} \end{array}\right]\;[\mathrm{by}\;\mathrm{Equation}\;5]$$

Let the demand be

$$D^{1}=\left[\begin{array}{ccc} \begin{array}{ccc} \;25 & 20 & 12 \end{array} & \begin{array}{ccc} 40 & 1 & 4 \end{array} & \begin{array}{ccc} 17 & 13 & \begin{array}{ccc} 4 & 5 & \begin{array}{cc} 2 & 1\;\;\; \end{array} \end{array} \end{array} \end{array}\right]$$

which is supposed to be satisfied by a schematic representation based on the priority and the Rh factor (refer to without substitution).

After the demand is satisfied by an exact match, the inventory levels of SDPs and RDPs are as follows:

$$X^{1}=\;\begin{array}{c} O-\\ O+\\ A_{1}-\\ A_{1}+\\ A_{2}-\\ A_{2}+\\ B-\\ B+\\ A_{1}B-\\ A_{1}B+\\ A_{2}B-\\ A_{2}B+ \end{array}\;\left[\begin{array}{ccccc} 0 & 0 & 0 & 0 & 0\\ 0 & 0 & 0 & 0 & 0\\ 0 & 0 & 0 & 0 & 0\\ 0 & 0 & 0 & 0 & 0\\ 1 & 0 & 0 & 0 & 0\\ 0 & 0 & 0 & 0 & 0\\ 0 & 0 & 0 & 0 & 0\\ 0 & 0 & 0 & 0 & 0\\ 0 & 2 & 3 & 1 & 0\\ 4 & 4 & 4 & 1 & 0\\ 2 & 0 & 0 & 0 & 0\\ 1 & 1 & 0 & 0 & 0 \end{array}\right]$$

$$Y^{1}=\;\begin{array}{c} O-\\ O+\\ A_{1}-\\ A_{1}+\\ A_{2}-\\ A_{2}+\\ B-\\ B+\\ A_{1}B-\\ A_{1}B+\\ A_{2}B-\\ A_{2}B+ \end{array}\;\left[\begin{array}{ccccc} 0 & 0 & 0 & 0 & 0\\ 0 & 0 & 0 & 0 & 0\\ 10 & 1 & 2 & 0 & 0\\ 0 & 0 & 0 & 0 & 0\\ 0 & 0 & 0 & 0 & 0\\ 0 & 0 & 0 & 0 & 0\\ 0 & 8 & 0 & 0 & 0\\ 0 & 0 & 0 & 0 & 0\\ 4 & 1 & 0 & 0 & 0\\ 0 & 0 & 0 & 1 & 0\\ 0 & 1 & 0 & 0 & 0\\ 0 & 0 & 0 & 0 & 0 \end{array}\right]$$

After it is satisfied without substitution, the resulting backlog is as follows:

$$BL^{1}=\left[\begin{array}{ccc} \begin{array}{ccc} \;\;5 & 5 & 0 \end{array} & \begin{array}{ccc} 10 & 0 & 1 \end{array} & \begin{array}{ccc} 0 & 1 & \begin{array}{ccc} 0 & 0 & \begin{array}{cc} 0 & 0\;\;\; \end{array} \end{array} \end{array} \end{array}\right]$$

**Figure 6:**
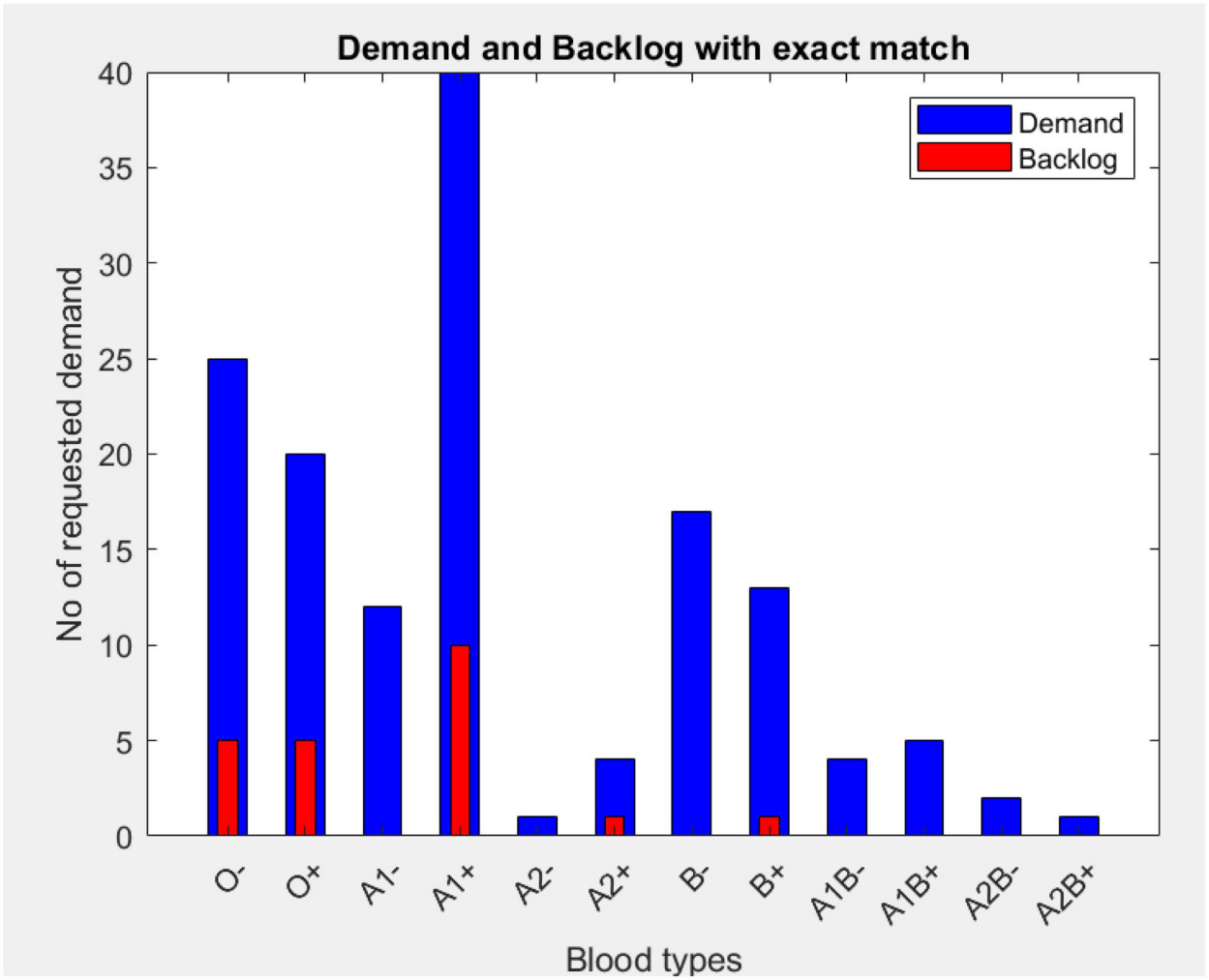
Demand satisfied with exact match.

Figure 6 represents the comparison of the demand vector $D^{1}$ and the backlog without substitution $BL^{1}$ was performed using MATLAB to illustrate the shortage in this particular case.

**Figure 7:**
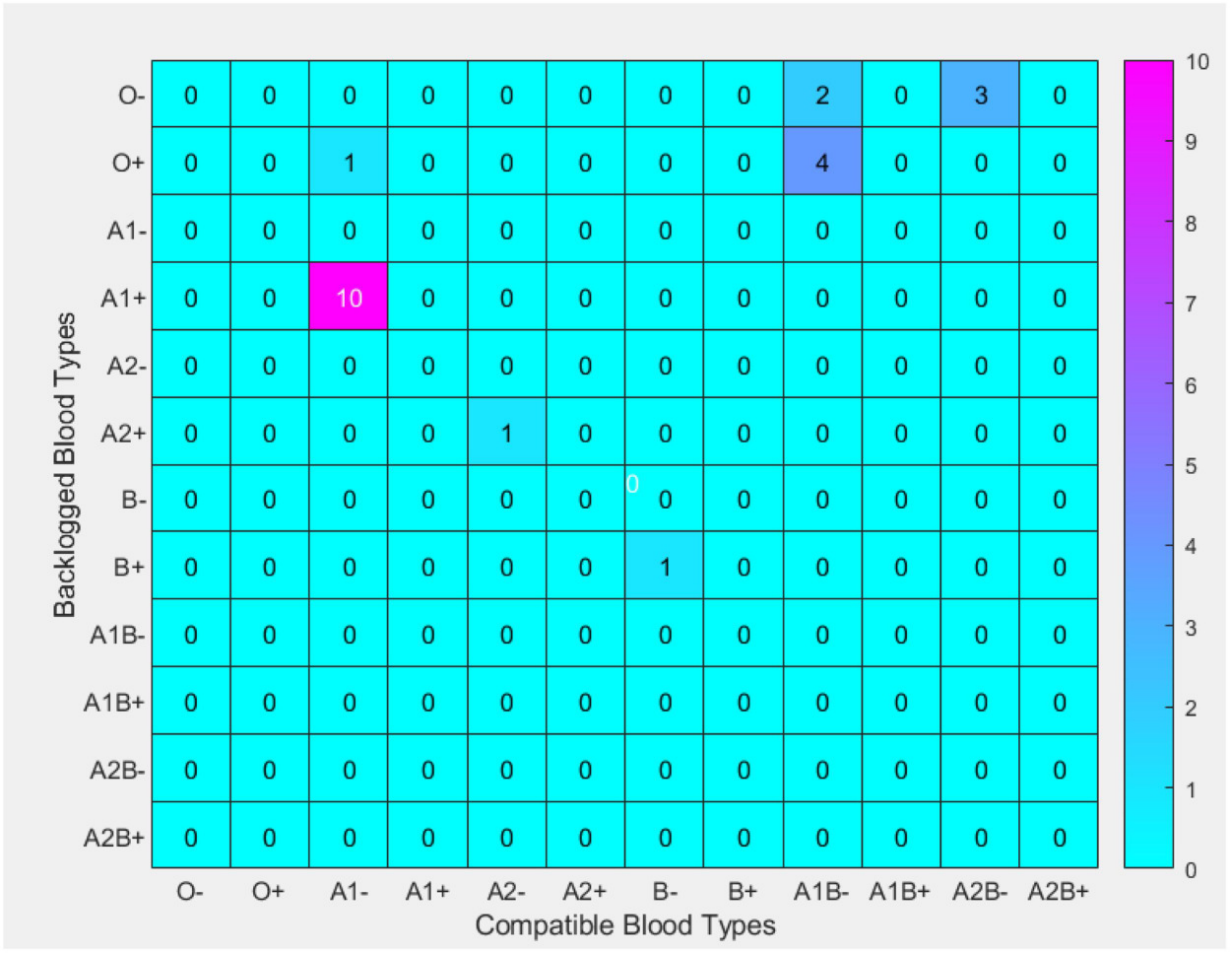
Demand satisfied with compatible substitution.

If the platelet bag only adheres to an exact match, it can result in shortages on particular days. If the platelet bag is unavailable, then cross-matching and $A_{1}A_{2}BO$ substitution are performed for the blood types O−, O+, $A_{1}+$, $A_{2}+$, and B+. Based on the preference table, donor availability, and inventory stock, the cross-matching substitution is selected, as outlined in Table 8 and Section 3.4.2. As shown in Figure 7, using MATLAB, this cross-matching allocation within the $A_{1}A_{2}BO$ system approach reduces the overall demand backlog by 15.28%. By optimizing inventory use and adhering to proper order management, the model successfully mitigates a 100% shortage at the given demand level, ultimately providing the highest service level.

**Table 8:** Cross-matching substitution for the backlogged platelets.

Backlog	Available donors	SDP	RDP
O− (5 units)	$A_{1}-,\;A_{2}-,\;B-,\;A_{1}B-,\;A_{2}B-$	$A_{1}B{-}^{\left(2\right)}$ (2 units)	-
		$A_{2}B{-}^{\left(3\right)}$ (1 unit)	
		$A_{2}B-$ (2 units)	
O+ (5 units)	All blood groups	-	$A_{1}B-$ (4 units)
			$A_{1}-(1$ unit)
$A_{1}+$ (10 units)	$A_{1}-,\;A_{1}B+,\;A_{1}B-$	-	$A_{1}-$ (10 units)
$A_{2}+\;$(1 unit)	$A_{2}-,\;A_{2}B+,\;A_{2}B-$	$A_{2}-$ (1 unit)	-
B+ (1 unit)	$B-,\;A_{1}B+,\;A_{1}B-,\;A_{2}B+,\;A_{2}B-$	-	*B−* (1 unit)

$k^{*}$, where * indicates the age of the product.

After the demand is satisfied without and with substitution, the shortage of the first week is,

$$S^{1}=\left[\begin{array}{ccc} \begin{array}{ccc} \;0 & 0 & 0 \end{array} & \begin{array}{ccc} 0 & 0 & 0 \end{array} & \begin{array}{ccc} 0 & 0 & \begin{array}{ccc} 0 & 0 & \begin{array}{cc} 0 & 0\;\;\; \end{array} \end{array} \end{array} \end{array}\right]$$

After the backlog was addressed through substitution, the inventory level of SDPs was adjusted as follows:

$$X^{1}=\;\begin{array}{c} O-\\ O+\\ A_{1}-\\ A_{1}+\\ A_{2}-\\ A_{2}+\\ B-\\ B+\\ A_{1}B-\\ A_{1}B+\\ A_{2}B-\\ A_{2}B+ \end{array}\;\left[\begin{array}{ccccc} 0 & 0 & 0 & 0 & 0\\ 0 & 0 & 0 & 0 & 0\\ 0 & 0 & 0 & 0 & 0\\ 0 & 0 & 0 & 0 & 0\\ 0 & 0 & 0 & 0 & 0\\ 0 & 0 & 0 & 0 & 0\\ 0 & 0 & 0 & 0 & 0\\ 0 & 0 & 0 & 0 & 0\\ 0 & 0 & 2 & 1 & 0\\ 4 & 4 & 4 & 1 & 0\\ 0 & 0 & 0 & 0 & 0\\ 1 & 1 & 0 & 0 & 0 \end{array}\right][\mathrm{by}\;\mathrm{Equation}\;30]$$

After the backlog was addressed through substitution, the inventory level of RDPs was adjusted as follows:

$$Y^{1}=\;\begin{array}{c} O-\\ O+\\ A_{1}-\\ A_{1}+\\ A_{2}-\\ A_{2}+\\ B-\\ B+\\ A_{1}B-\\ A_{1}B+\\ A_{2}B-\\ A_{2}B+ \end{array}\;\left[\begin{array}{ccccc} 0 & 0 & 0 & 0 & 0\\ 0 & 0 & 0 & 0 & 0\\ 0 & 1 & 2 & 0 & 0\\ 0 & 0 & 0 & 0 & 0\\ 0 & 0 & 0 & 0 & 0\\ 0 & 0 & 0 & 0 & 0\\ 0 & 7 & 0 & 0 & 0\\ 0 & 0 & 0 & 0 & 0\\ 4 & 1 & 0 & 0 & 0\\ 0 & 0 & 0 & 1 & 0\\ 0 & 1 & 0 & 0 & 0\\ 0 & 0 & 0 & 0 & 0 \end{array}\right][\mathrm{by}\;\mathrm{Equation}\;31]$$

Once the backlog is addressed, the final row of the state matrix becomes obsolete, rendering SDPs outdated for day 1,

$$SO^{1}=\left[\begin{array}{ccc} \begin{array}{ccc} \;\;0 & 0 & 0 \end{array} & \begin{array}{ccc} 0 & 0 & 0 \end{array} & \begin{array}{ccc} 0 & 0 & \begin{array}{ccc} 0 & 0 & \begin{array}{cc} 0 & 0\;\;\; \end{array} \end{array} \end{array} \end{array}\right]$$

RDPs were outdated on day 1,

$$RO^{1}=\left[\begin{array}{ccc} \begin{array}{ccc} \;0 & 0 & 0 \end{array} & \begin{array}{ccc} 0 & 0 & 0 \end{array} & \begin{array}{ccc} 0 & 0 & \begin{array}{ccc} 0 & 0 & \begin{array}{cc} 0 & 0\;\;\; \end{array} \end{array} \end{array} \end{array}\right]$$

The overall outdated result on day 1 is as follows:

$$O^{1}=\left[\begin{array}{ccc} \begin{array}{ccc} \;\;0 & 0 & 0 \end{array} & \begin{array}{ccc} 0 & 0 & 0 \end{array} & \begin{array}{ccc} 0 & 0 & \begin{array}{ccc} 0 & 0 & \begin{array}{cc} 0 & 0\;\;\; \end{array} \end{array} \end{array} \end{array}\right]$$

The replenishment will bring the inventory to the pre-established level. $V_{k}^{1}$ considers all ages; based on the order received from SDPs, the RDP level is determined using the order-up-to-level.

i.e.,RDPinventorylevel=orderuptolevel−SDPinventorylevel

$$Y^{1}\;=\;V_{k}^{1}\;-\;X^{1}$$

Then the total inventory level is equal to

$$Z^{1}(1)=\left[\begin{array}{ccc} \begin{array}{ccc} 28 & 35 & 25 \end{array} & 45 & \begin{array}{ccc} 2 & 5 & 2\begin{array}{ccc} 5 & 20 & \begin{array}{cc} 15 & \begin{array}{ccc} 20 & 5 & 3\;\;\;\; \end{array} \end{array} \end{array} \end{array} \end{array}\right]$$

$$=L^{1}$$

Next, the inventory remaining at the end of day 1 will be carried forward and available at the start of day 2, subject to updates: $Z^{1+1}\;=\;Z^{1}$

The graphs (see Figures 6, 7) show that the shortage of blood on a given day is significantly reduced by implementing the allocation procedure (see Figures 4, 5). Hence, an adequate service level was achieved with this issuing policy. The detailed methods and results of this numerical example are discussed in the discussion section. In the numerical example, the order quantity is determined based on the $A_{1}A_{2}BO$ preference table, with orders filled according to donor-recipient compatibility. Initial inventory levels for SDP and RDP are assumed based on platelet compatibility within the $A_{1}A_{2}BO$ system (refer to Tables 1, 3, 4), while also considering the phenotype frequency of the population (refer to Figure 1). The initial platelet demand on a given day is met through exact matches; however, subsequent backlogs result in a 22-unit shortage, posing critical risks to patient health. By leveraging the $A_{1}A_{2}BO$ substitution table, the product preference table, the age mismatch table, and the backlog for blood types $O-,\;O+,\;A_{1}+,\;A_{2}+,$ and B+ are efficiently addressed in accordance with the compatible preferences outlined in Table 1. In this substitution approach, the demand for blood type O− is influenced by the age of the platelets; the possible compatible donors are $A_{1}-,\;A_{2}-,\;B-,A_{1}B-$ and $A_{2}B-$. Upon inventory management's analysis of all demand backlogs in the remaining blood groups, priority is given to addressing backlogs for $A_{1}+,\;A_{2}+,$ and B+ blood types by utilizing $A_{1}-,\;A_{2}-,$ and B− units, respectively. Initially, the demand for the rare blood type $A_{2}+$ (1 unit) is satisfied with fresh platelets from $A_{2}-$ donors sourced from SDPs. The backlog demand for O− blood type, totaling 5 units, is subsequently addressed by utilizing platelets from the subtype of the universal donor of platelets.

- 2 units of $A_{1}B-$ platelets are utilized, with a 2-day age and a 3-day shelf-life.
- 1 unit of $A_{2}B-$ platelets are used, with an age of 3 days and balance shelf life of 2 days.
- 1 unit of $A_{2}B-\;$ platelets are utilized, with an age of 3 days and 2 days of balance shelf life.
- 2 units of $A_{2}B-$ fresh platelets are sourced from the SDP, with a 5-day balance shelf-life, ensuring operational performance by minimizing the risk of RBC contamination compared with RDP allocations.

This strategy ensures operational performance by minimizing the risk of RBC contamination, as SDP allocations are preferred over RDPs. As illustrated in Figure 5, the backlog demand for 5 units of O+ is contingent on age and is addressed by utilizing fresh platelets, along with 4 units of $A_{1}B-$ and 1 unit of $\;A_{1}-\;$ obtained from RDP. Similarly, the age-dependent backlogs for $A_{1}+$ (10 units) and B+ (1 unit) are satisfied by utilizing 10 units of $A_{1}-$ and 1 unit of B−, respectively, sourced from fresh platelets obtained from RDPs.

### 4.3 Simulation

The simulation model was developed using Arena to represent the platelet issuing system. The entity flow follows a step-by-step process that starts with demand generation, allocation decisions, and inventory updates. Platelet inventories are classified as separate resources for SDP and RDP units, categorized as fresh and ordinary, with finite capacities. Allocation decisions follow a hierarchical structure, starting with exact matching, then by product switching, compatibility-based cross-matching, and substitution rules. Queue disciplines are implemented based on demand characteristics. Age-dependent demand follows the FUFU policy, while age-independent demand follows the OUFU policy. Hybrid FUFU/OUFU switching is implemented using decision logic and attribute-based routing within the Arena environment.

**Figure 8:**
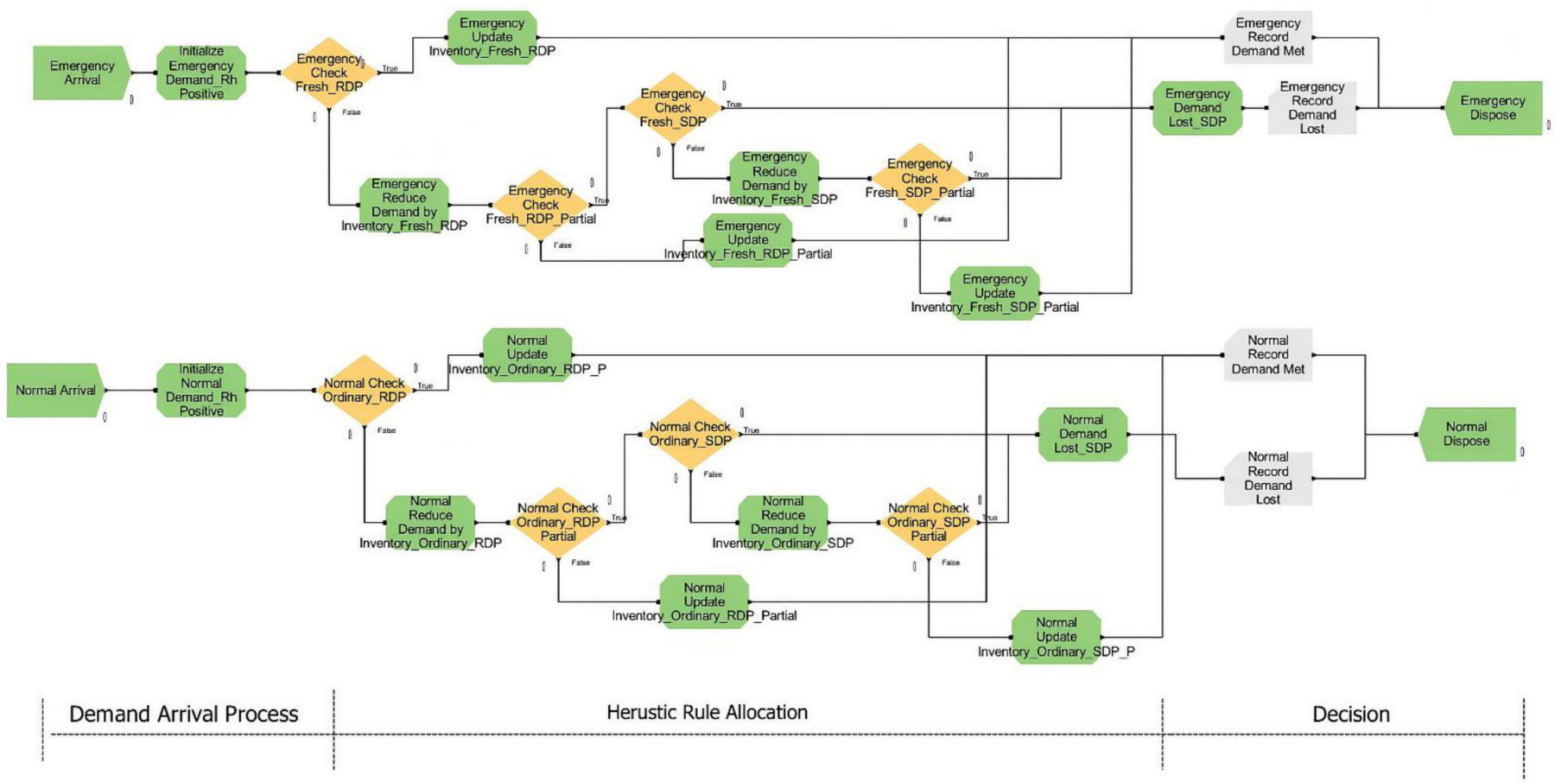
ARENA environment of exact age match, product mismatch, and partial backlog allocation.

To evaluate the effectiveness of the proposed model simulation experiments were conducted across six distinct operational scenarios. Each scenario reflects a combination of patient blood subtype, product compatibility, backlog status, and allocation logic. The key performance metrics assessed include the service level, waste rate, substitution rate, and allocation precision. The arena environment of this scenario is shown in Figure 8. The scenarios are described as follows:

*Scenario 1: (S1)* $A_{1}+$ *Demand—Exact blood type, exact age match, exact product, full backlogging:*

This scenario represents an idealized clinical condition wherein the patient's blood group ($A_{1}+$, fresh or ordinary) and requested platelet product type (SDPs or RDPs) are exactly matched. A full backlogging policy is applied; that is, all transfusion requests are deferred until a fully compatible unit becomes available.

*Scenario 2: (S2)* $A_{1}+$ *Demand—Exact blood type, exact age match, exact product, partial backlogging:*

This scenario maintains strict compatibility in terms of blood group, age, and product type, as in Scenario 1. However, a partial backlogging policy is implemented, simulating a more practical allocation strategy that prioritizes urgent cases.

*Scenario 3: (S3)* $A_{1}+$ *Demand—Exact blood type, exact age match, product mismatch, partial backlogging:*

Here, blood type and age compatibility are preserved, but product substitution between SDP and RDP is allowed when the exact product is unavailable. Partial backlogging is employed to handle unmet demand in a semiflexible manner.

*Scenario 4: (S4)* $A_{2}+$ *Demand—Platelet request in the ABO system, exact match:*

This scenario applies a conventional ABO-based allocation policy that does not differentiate between the $A_{1}\;$ and $A_{2}$ subtypes. A patient with a blood group $A_{2}+$ receives platelet products based solely on ABO compatibility, reflecting current standard practices.

*Scenario 5: (S5)* $A_{2}+$ *Demand—Platelet request in the* $A_{1}A_{2}BO$ *system, exact blood type, exact age match, product mismatch, partial backlogging:*

This case implements the proposed $A_{1}A_{2}BO$ allocation framework that precisely matches blood group and age while allowing substitution of platelet product types. Partial backlogging is applied, representing a flexible yet operationally cautious issuing technique.

*Scenario 6: (S6)* $A_{2}+$ *Demand—Platelet request in the ABO system, compatible blood type, exact age match, product mismatch, partial backlogging:*

This scenario evaluates the ABO-based allocation strategy for a patient with blood group $A_{2}+$, where compatibility is assessed at the ABO level because of the lack of subtype differentiation. Although we prioritize age matching, the current unavailability of the desired product requires us to explore alternatives. Partial backlogging is applied to manage unmet demand in an operationally adaptive manner.

PIM issuing policy with performance measured across six operational scenarios and four inventory settings (a, b, c, d). These cases represent low to high inventory conditions. Scenarios 4 and 6 are considered baseline reference cases representing conventional ABO-based allocation within the simulation framework, with limited to specific cases. Results are based on 30 simulation replications and reported with 95% confidence intervals, a 24-hour warm-up period and a replication length of 720 h. The model's effectiveness is assessed using the key performance indicators (KPIs) to minimize shortages and reduce waste, while striving to achieve the highest possible service levels. The dual focus on scenario-level design and attained performance enables a comprehensive evaluation of both the theoretical robustness and practical feasibility of the issuing strategy. Notably, the issuing model does not rely solely on the current inventory state but incorporates demand classification, phenotype frequency, and anticipated next-day needs. This demand-driven framework ensures that ordering and allocation decisions account for uncertainty and allocation constraints. The target inventory levels, derived from Table 9, vary by phenotype and blood group subtype and are particularly critical for groups $A_{2}$ with lower frequency. The simulation evaluates each scenario under varying inventory conditions to identify the most operationally favorable configuration. The demand distributions are derived from a 15-day observational dataset. This duration represents approximately three complete inventory cycles, considering the typical 5-day shelf life of platelet products. Because the platelet inventory operates under short-cycle dynamics, this period provides a representative snapshot of demand and usage patterns for modeling. However, the limited duration may not fully capture rare blood group occurrences, and this is acknowledged as a limitation of the study.

**Table 9:** Blood group demand and inventory data from the regional blood bank (RBB) for 15 days.

S. No	Blood group	RBB SDP demand			RBB RDP demand			Phenotype frequency (%)	Inventory capacity	
		Distribution	Mean	SD	Distribution	Mean	SD		SDP	RDP
1	O−	Poisson	0.0667	0.258	–	–	–	3.96	1	2
2	O+	Poisson	0.4	0.632	Beta	4.93	2.69	40.09	4	20
3	$A_{1}-$	–	–	–	–	–	–	2.78	–	1
4	$A_{1}+$	Poisson	0.4	0.632	Beta	1.8	1.57	24.12	2	12
5	$A_{2}-$	–	–	–	–	–	–	0.69	–	–
6	$A_{2}+$	–	–	–	–	–	–	6.03	1	3
7	B−	Weibull	0.133	0.352	Beta	1.33	0.976	1.33	1	1
8	B+	Exponential	0.333	0.617	Normal	3.53	1.64	15.73	2	8
9	$A_{1}B-$	–	–	–	–	–	–	0.41	–	2
10	$A_{1}B+$	Weibull	0.2	0.414	Lognormal	0.333	0.724	3.58	–	2
11	$A_{2}B-$	–	–	–	–	–	–	0.1	–	–
12	$A_{2}B+$	–	–	–	–	–	–	1.16	–	1

#### 4.3.1 Simulation-based issuing process (Algorithmic Pseudo code)

Initialize:

 Set *t* = 0

 Initialize inventory for all blood groups (SDP, RDP, age)

 Initialize performance metrics:

  Total Demand = 0

  Satisfied Demand = 0

  Shortage = 0

  Wastage = 0

While *t* < Simulation End Time do:

 Step 1: Generate Demand

  Generate patient demand based on interarrival time (EXPO(*λ*))

  Identify:

   Blood group (A1, A2, B, O, AB)

   Rh type (±)

   Demand type (Age-dependent/Age-independent)

   Demand Quantity (Set the distribution)

   Product type (SDP/RDP)

  Total Demand = Total Demand + 1

 Step 2: Exact Match Allocation

  If exact blood group and product available then

   Allocate using:

    If Age-dependent → FUFU

    Else → OUFU

   Update inventory

   Satisfied Demand = Satisfied Demand + 1

   Go to Step 6

  End If

 Step 3: Product Switching (SDP ↔ RDP)

  If alternate product available then

   Allocate using same priority rule

   Update inventory

   Satisfied Demand = Satisfied Demand + 1

   Go to Step 6

  End If

 Step 4: Cross-Matching (ABO Compatibility)

  If compatible blood group available then

   Allocate using priority rule

   Update inventory

   Satisfied Demand = Satisfied Demand + 1

   Go to Step 6

  End If

 Step 5: Substitution Rule

  If any compatible + product substitution available then

   Allocate

   Update inventory

   Satisfied Demand = Satisfied Demand + 1

  Else

   Shortage = Shortage + 1

  End If

 Step 6: Inventory Aging

  Increase age of all platelet units

  Remove expired units (age > shelf life)

  Wastage = Wastage + expired units

 Step 7: Advance Time

  Update simulation clock *t*

End While

Record Compute Performance Metrics:

 Service Level = Satisfied Demand/Total Demand

 Total Shortage Rate = Shortage/Total Demand

 Total Wastage Rate = Wastage/Total Inventory

End

#### 4.3.2 Evaluation of $A_{1}+$ blood type scenarios

**Figure 9:**
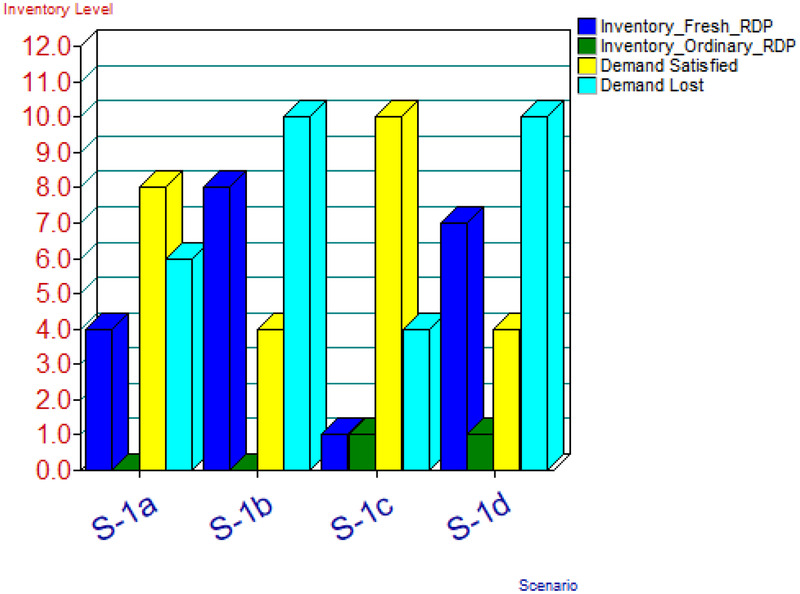
Comparison of scenario 1 with four different inventory level settings for blood type $A_{1}+$ demand.

**Figure 10:**
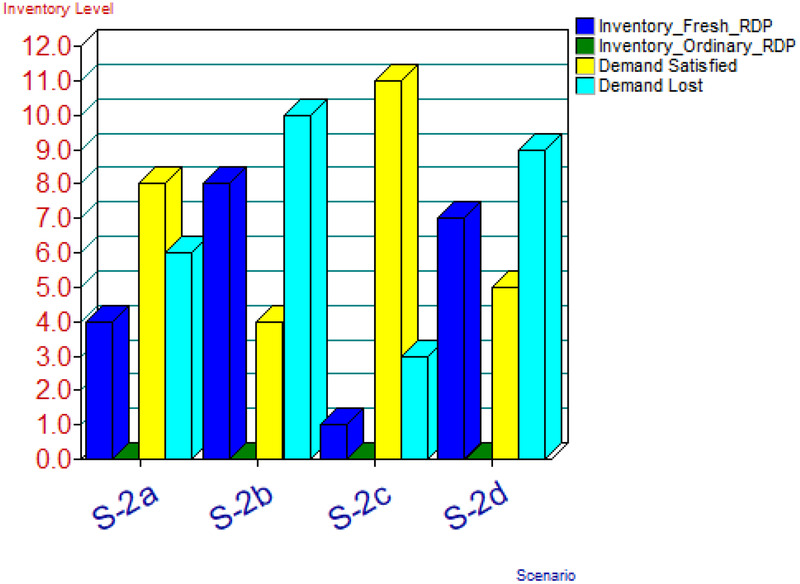
Comparison of scenario 2 with four different inventory level settings for blood type $A_{1}+$ demand.

**Figure 11:**
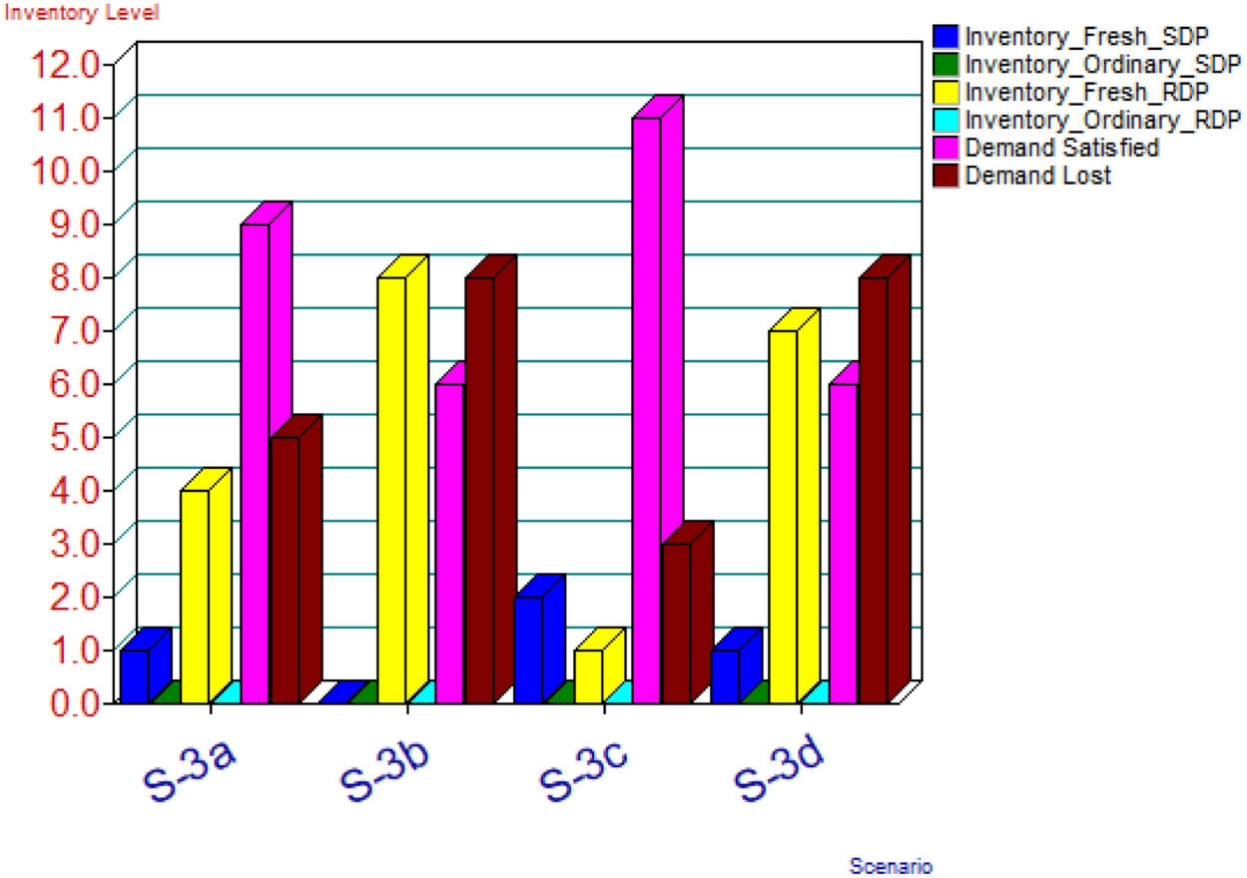
Comparison of scenario 3 with four different inventory level settings for blood type $A_{1}+$ demand.

The $A_{1}+$ demand are detailed in Table 10 and Figures 9–11. In Scenario 1 (exact match with full backlog), configuration S1-c achieved the highest service level across all inventory levels, indicating available inventory and patient needs. Similarly, in Scenario 2 (exact match with partial backlog), S2-c outperformed S2-a, S2-b, and S2-d. The scenario service level (theoretical maximum) for S2-c was 99%, while the attained service level (simulation result) was 78.57%. It shows the importance of partial backlogging, which improves allocation flexibility with operational performance, in Rh-positive cases where substitution is acceptable. In Scenario 3 (product mismatch with partial backlog), configuration S3-c also achieved a service level of 78.57%. The inventory settings S3-a, S3-b, and S3-d showed greater variations in outcomes, indicating greater sensitivity to inventory positioning under mismatch conditions. The trade-offs here are evident: while product substitution enables continued service, its success depends heavily on the structure of remaining stock and substitution logic. By comparing policy behavior (scenario service level) and the simulation outcomes (attained service level), S2-c emerges as the optimal configuration, offering an operationally feasible solution. The presence of partial backlog support, sufficient Rh + inventory, and age match conditions make it a balanced allocation under uncertain demand.

**Table 10:** $A_{1}+$ blood type scenario analysis.

Scenarios	Scenario service level %	Targeted inventory level				Remaining inventory level				Scenario results		Attained service level %	Average attained service level
		SDP F	SDP O	RDP F	RDP O	SDP F	SDP O	RDP F	RDP O	Demand satisfied	Demand lost		
S1-a	99.9	1	1	6	6	1	1	4	0	8	6	57.14	
S1-b	99.9	0	2	10	2	0	2	8	0	4	10	28.57	0.46 ± 0.42
S1-c	99.9	2	0	3	9	2	0	1	1	10	10	71.43	
S1-d	99.9	1	1	9	3	1	1	7	1	4	10	28.57	
S2-a	99	1	1	6	6	1	1	4	0	8	6	57.14	
S2-b	99	0	2	10	2	0	2	8	0	4	10	28.57	0.49 ± 0.89
S2-c	99	2	0	3	9	2	0	1	0	11	3	78.57	
S2-d	99	1	1	9	3	1	1	7	0	5	9	35.71	
S3-a	98	1	1	6	6	1	0	4	0	9	5	64.29	
S3-b	98	0	2	10	2	0	0	8	0	6	8	42.86	0.57 ± 0.14
S3-c	98	2	0	3	9	2	0	1	0	11	3	78.57	
S3-d	98	1	1	9	3	1	0	7	0	6	8	42.86	

F, fresh; O, ordinary.

#### 4.3.3 Evaluation of $A_{2}+$ blood type scenarios

**Figure 12:**
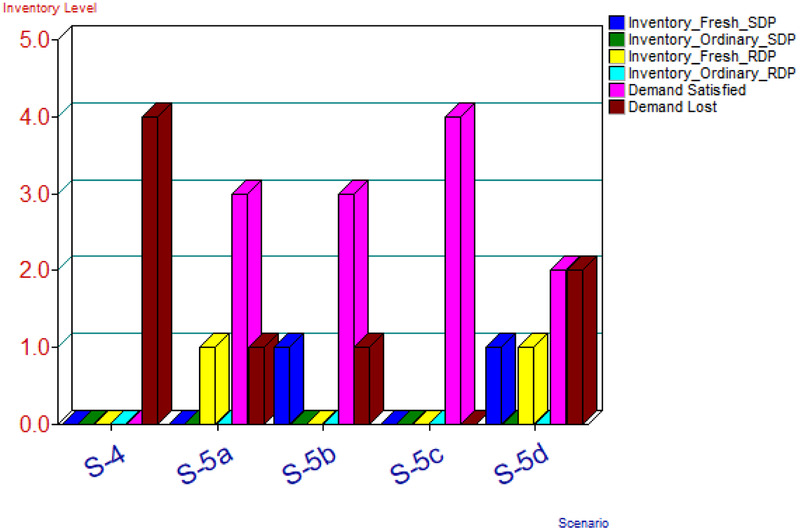
Comparison of scenario 4 with four different inventory level settings for blood type $A_{2}+$ demand in the ABO system.

**Figure 13:**
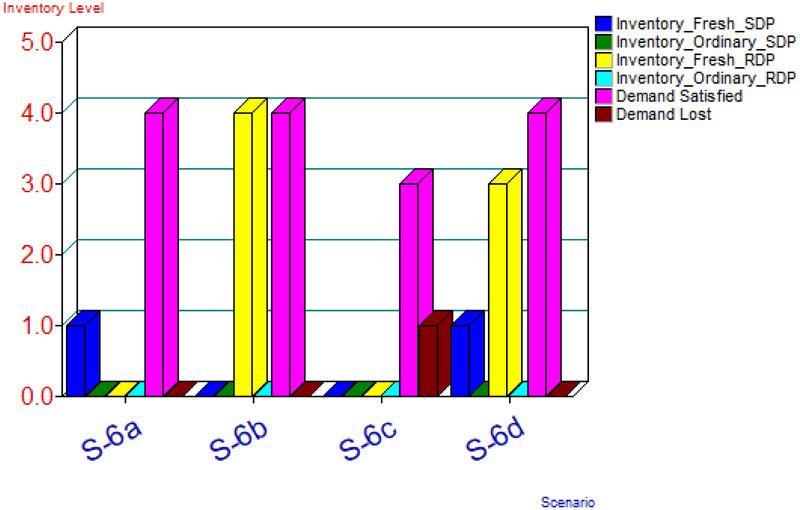
Comparison of scenario 6 with four different inventory level settings for blood type $A_{2}+$ demand in the $A_{1}A_{2}BO$ system.

**Figure 14:**
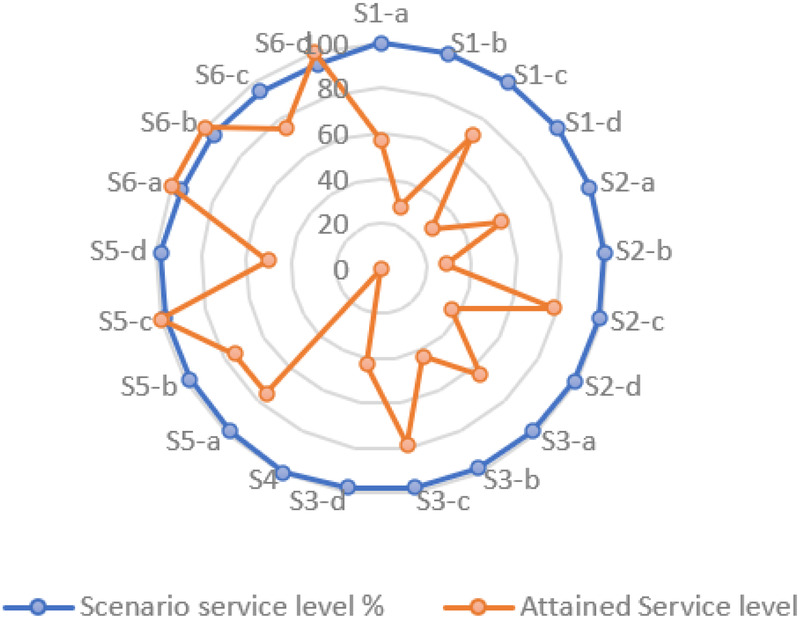
Juxtaposition of the service level.

A comparative evaluation was conducted between the traditional ABO system and the proposed system. $A_{1}A_{2}BO$ classification to assess their effectiveness in handling $A_{2}+$ demand. Scenario 4 (ABO-based allocation) is treated as the baseline policy for comparison with the proposed $A_{1}A_{2}BO$ model. As shown in Table 11 and Figure 12, under the ABO system, no inventory was available to fulfill an exact-match request for $A_{2}+$, resulting in a scenario service level of 99% but an attained product match rate of 0%. This highlights the fundamental limitation of ABO-only policies in supporting subtype-specific allocation. Whereas the proposed $A_{1}A_{2}BO$ system (Scenario 5) addresses this gap by explicitly accounting for $A_{1}/A_{2}$ differentiation. Among the four inventory configurations (S5-a to S5-d), S5-c achieved the highest performance, with a scenario service level of 98% and an attained service level of 100%. These results validate the operational relevance of incorporating subtypes into policy issuance, particularly for demand categories with low phenotype frequency. To further explore strategies, Scenario 6 was introduced to simulate $A_{2}+$ demand fulfillment using the residual $A_{1}+$ inventory under the ABO system. As illustrated in Figure 13, configurations S6-a, S6-b, and S6-d attained 100% service levels due to the sufficient compatible stock. However, S6-c led to near depletion of the $A_{1}+$ inventory. This shows a significant operational risk: if a subsequent $A_{1}+$ demand occurs, the blood bank may be unable to fulfill it, even with product substitution. Such a scenario would compromise the operational performance and responsiveness of the issuing policy. Moreover, under age-independent demand conditions, none of the configurations in S6 (except S6-c) retained sufficient SDP or RDP units for $A_{1}+$. In those cases, the feasible option would be to allocate fresh SDPs or RDPs, which would significantly increase operational costs. From Figure 14, S5-c emerges as the optimal scenario, balancing compatibility, inventory utilization, and operational performance. From the analysis, both the scenario service level and the attained service level were reported to support decision-making. This dual evaluation enables inventory managers to differentiate between theoretically robust policies and those that hold under practical constraints.

**Table 11:** $A_{2}+$ blood type scenario analysis.

Scenarios	Scenario service level %	Targeted inventory level				Remaining inventory level				Scenario results		Attained service level %	Average attained service level
		SDP F	SDP O	RDP F	RDP O	SDP F	SDP O	RDP F	RDP O	Demand satisfied	Demand lost		
S4	99.9	0	0	0	0	0	0	0	0	0	4	0	0
S5-a	98	0	1	2	1	0	0	1	0	3	1	75	
S5-b	98	1	0	1	2	1	0	0	0	3	1	75	
S5-c	98	0	1	1	2	0	0	0	0	4	0	100	75 ± 0.56
S5-d	98	1	0	2	1	1	0	1	0	2	2	50	
Scenarios	$A_{1}\;$ Remaining inventory from S3					Remaining inventory level				Scenario results			
S6-a	95	1	0	4	0	1	0	0	0	4	0	100	
S6-b	95	0	0	8	0	0	0	4	0	4	0	100	
S6-c	95	2	0	1	0	0	0	0	0	3	1	75	93 ± 0.75
S6-d	95	1	0	7	0	1	0	3	0	4	0	100	

F, fresh; O, ordinary.

## 5 Discussion

The cross-matching substitution for allocation has led to a notable reduction in the shortage for the twelve blood types on that specific day. When no stock is available that meets compatible preferences, the unmet demand is categorized as a shortage, enabling a systematic evaluation of allocation performance. The operational feasibility of incorporating dynamic allocation logic into inventory settings. These findings emphasize the importance of aligning inventory levels with phenotype frequency to support efficient allocation. The proposed issuing policy incorporates age-based prioritization of platelet products, particularly the use of RDP units with a minimum remaining shelf life, to balance inventory utilization and demand satisfaction. While such assumptions of the model improve allocation efficiency in the simulated setting, their practical implementation would require careful consideration of real-world operational constraints. The analysis further highlights the limitations of conventional ABO-based allocation, particularly in handling $A_{2}$ demand without subtype classification. The integration of the $A_{1}A_{2}BO$ classification enables more structured compatibility handling and improves inventory utilization under constrained conditions. In this context, a subtype-aware allocation policy reduces the reliance on broad compatibility substitutions and enhances allocation precision within the proposed system. Enhancing service levels in this context is crucial for promoting long-term sustainability in healthcare. Furthermore, this model provides a comprehensive analysis of donor-recipient compatibility in blood types, with a numerical example comparing red blood cell and platelet donor-recipient interactions. In cases where platelets are unavailable or when cross-matching is not appropriate, the system moves to the following preferred blood type, as outlined in the $A_{1}A_{2}BO$ compatibility chart (refer to Table 1).

The results also indicate that inventory utilization can be improved by incorporating shelf-life-based allocation strategies under heterogeneous demand conditions. The proposed allocation framework considers both compatibility requirements and overall inventory conditions to support balanced utilization across all blood groups, ensuring fair and equitable resource distribution while addressing patient-specific needs. These findings highlight the need to maintain consistent service levels while balancing resource utilization within a simulated environment. While sensitivity analyses have been conducted for inventory levels and shelf-life conditions, other parameters, such as switching thresholds, Rh preference weights, and phenotype distributions, may also influence system performance. The proposed framework allows these parameters to be adjusted, and their detailed analysis is considered for future research. Future work will include the development of a comprehensive baseline scenario to enable full-system comparison with conventional allocation strategies. To substantiate the proposed policy, a comprehensive set of research questions was crafted to test the PIM policy's hypotheses. The responses were gathered from clinical experts to validate the findings of the model. Detailed research questions, presented as statements, are included in the Supplementary Material. Notably, the findings are based on simulation experiments and numerical illustrations and do not incorporate patient-level clinical outcomes. The primary validation of the model is based on simulation analysis, with expert feedback serving as supportive evidence. However, further validation using real-world clinical data is needed to assess its practical applicability.

### 5.1 Adaptability of the proposed model

The distribution of blood types varies significantly across geographic regions because genetic, ethnic, and demographic factors directly impact BIM. A comprehensive classification of the *n* population, based on region-based and religion-based surveys, is provided in (23, 31, 33, 58). This research highlights the importance of integrating this classification into blood bank management systems. Fewer studies have been conducted on $A_{1}A_{2}BO$ blood groups. An analysis of 20,864 donors over two years revealed the following prevalence rates: $A_{1}+\;(24.86\text{\%}),\;A_{2}+\;(0.3\text{\%}),\;A_{1}B+\;(7.13\text{\%}),\;A_{2}B+\;(0.84\text{\%}),$ $A_{1}-\;(1.05\text{\%}),\;A_{1}B-\;(0.21\text{\%}),\;A_{2}B-\;(0.014\text{\%}),$ and $A_{2}-$ (0.004%) (23).

To address challenges in blood type distribution, an adaptive allocation policy is proposed that allows blood banks to adjust age-based issuing preferences, Rh compatibility rules, and subtype considerations based on geographic blood type availability. Such flexibility supports real-world applicability. For instance, in regions where AB− blood is scarce, priority stocking of SDP from AB− donors may be necessary to prevent shortages. Conversely, in areas with higher O-type prevalence, the model can prioritize O (high population frequency) or AB (universal donors in platelets) platelet allocation while ensuring cross-matching compatibility to prevent waste. The proposed $A_{1}A_{2}BO$ system, which represents the world population (Figure 1), requires further modification to be incorporated into blood bank systems. A modified $A_{1}A_{2}BO$ system is proposed, which reduces the number of blood types from 12 to 10 by ignoring rare Rh-negative blood types $A_{2}-$ and $A_{2}B-\;$ due to the limited availability of genotype populations. Future research will focus on the modified $A_{1}A_{2}BO\;$ system, which aims to improve the efficiency of blood bank inventory management. The proposed patient-centric policy aligns with the 2025 AABB & International Collaboration for Transfusion Medicine Guidelines (ICTMG) clinical guidelines, supporting compatibility considerations in platelet transfusion practices (47). Future pilot implementations are required to assess practical applicability in real-world settings.

### 5.2 Managerial insights

- *Implementing a combination of FUFU and OUFU policies* within blood bank operations enables healthcare managers to maintain optimal inventory levels, reduce wastage from expired units, and ensure timely access to the appropriate supplies for patients. This strategic combination addresses fluctuating inventory demand while upholding safety standards.
- *Adopting this issuing policy* not only addresses existing inefficiencies within the system but also positions the blood bank to respond to future fluctuations in demand efficiently, ensuring consistent service quality without compromise.
- *The case study, is based on representative treatment scenarios and allocation strategies*, demonstrates the potential to enhance inventory performance and provides practical insights into the implementation of the proposed policy as a decision-support tool in blood bank operations.
- *Decision-support frameworks* can be integrated with existing blood bank information systems as a decision-support. Rule-based allocation logic allows modular implementation, where individual components such as compatibility checking, substitution rules, and priority policies can be incorporated progressively. The scalability and supports practical adoption in real-world settings under varying data availability and operational constraints.

### 5.3 Future scope of the research

Future implications for various sectors such as blood collection, hematology, and healthcare related technological advancements are represented by the integrated approach to PIM. The following avenues for future exploration and implementation are anticipated:

- Future clinical research will assess the applicability of platelet transfusion by applying the model and analyzing the CCI in transfused patients. This helps determine the system's ability to improve allocation performance.
- Clinical researchers or hematologists will evaluate the effectiveness of the current platelet transfusion system in comparison to the proposed issuing policy, particularly in terms of integrating the $A_{1}\;A_{2}\;BO$ system, which aims to improve compatibility and overall allocation effectiveness.
- The training of blood bank personnel can create complexity and errors in the system, whereas human error can be circumvented by utilizing technology-driven systems to automate workflows. e.g., automated workflow management systems, barcode scanning and tracking systems, data analytics, and reporting tools.
- Advancements in blood group systems technology, in conjunction with the integration of cross-matching preferences into the IIoT, have the potential to increase the efficiency of platelet allocation, reducing the time required for manual processes. This integration will give blood product service centers greater access to data and improved visibility, enabling better decision-making.
- Inventory management will allocate platelets to backlog demand requests through the ABO system, particularly in rare cases. Future research will continue to focus on addressing platelet shortages, optimizing inventory positions, and refining the allocation system to ensure the highest level of service.

Through these steps, this system will evolve, modernizing platelet inventory management and paving the way for more efficient healthcare delivery.

## 6 Conclusion

A simulation-based, patient-centric platelet issuing policy to enhance operational efficiency in blood bank management. The model incorporates critical clinical parameters, including blood subtype classification $\left(A_{1}/A_{2}\right)$, type of platelet products (SDPs and RDPs), and age-specific compatibility requirements, to maximize allocation under precision under heterogeneous demand. Although the current work does not include direct clinical trial data, the policy design is grounded in prior medical studies, informed by expert clinical input, and evaluated through simulation-based comparative analysis with existing allocation practices. The findings suggest that the proposed policy has the potential to improve inventory performance by reducing wastage and enhancing allocation precision.

The model supporting decision-making in platelet inventory management. It integrates domain knowledge with simulation-based analysis to evaluate allocation strategies under realistic constraints. The results should be interpreted as indicating operational feasibility within a simulated environment rather than clinical outcomes. While real-world platelet transfusion practices often involve flexible compatibility decisions due to inventory constraints, the proposed model adopts a structured policy to systematically evaluate allocation strategies under controlled conditions. Future work will focus on validation with real-world data, incorporation of operational constraints, and refinement of the allocation framework to support practical implementation.

Sustainability development goals 2023 3Good health and well-being.

## Data Availability

The original contributions presented in the study are included in the article/Supplementary Material, further inquiries can be directed to the corresponding author.
