# Supplementary material for: Heuristic platelet issuing policies for an integrated ABO blood bank system under heterogeneous demand: towards patient-centric management
Source: Front Health Serv. 2026 Jun 10;6:1813185. doi: 10.3389/frhs.2026.1813185 (PMC13290749; doi:10.3389/frhs.2026.1813185)
Supplement: Supplementary file 1 [file Datasheet1.pdf]

Appendix - Questionnaires

1. In clinical settings, managing and minimizing waste and shortages of blood products such as RBCs, platelets, and whole blood is crucial due to their perishability.
   a) Surgeon
   b) Blood bank technical supervisor
   c) Director of blood transfusion center
   d) **All of the above**
2. Hospital management and blood bank management both share the responsibility of assessing the age of blood products based on patient considerations.
   a) Surgeon
   b) Blood bank technical supervisor
   c) Director of blood transfusion center
   d) All of the above
3. Platelet contamination occurs during the separation process from whole blood, leading to potential post-transfusion reactions associated with platelets.
   a) Surgeon
   b) Blood bank technical supervisor
   c) **Director of blood transfusion centre**
   d) All of the above
4. Considering blood types is significant in preventing transfusion-related reactions, but it's also essential to consider the subtypes for comprehensive safety measures.
   a) Surgeon
   b) Blood bank technical supervisor
   c) Director of blood transfusion center
   d) All of the above

5. Upgrading the ABO system to A1A2BO could enhance the identification of post-transfusion reactions through clinical studies. For this upgraded system, implementing a proper allocation policy is necessary to improve its significance in both clinical settings and blood banks.
   a) Surgeon
   b) Blood bank technical supervisor
   c) Director of blood transfusion center
   d) All of the above
6. Ignoring specific subtypes during transfusions can lead to post-transfusion reactions, potentially resulting in mortality or organ failure.
   a) Surgeon
   b) Blood bank technical supervisor
   c) Director of blood transfusion center
   d) All of the above
7. Upgrading the blood group system in blood banks has indeed enhanced the quality of service, efficiency, accuracy, and patient outcomes.
   a) **Surgeon**
   b) Blood bank technical supervisor

c) **Director of blood transfusion center**
d) All of the above

8. Patient-centric demand is crucial for enhancing the accuracy of transfusions.
a) Surgeon
b) Blood bank technical supervisor
c) Director of blood transfusion center
d) **All of the above**

Research Questions

With the help of these questions, blood banking and transfusion treatment will advance! Explore the key strategies employed by blood banks to mitigate risks and ensure transfusion safety, and discover the latest advancements in the field. With a focus on upgrading the current blood bank system, this questionnaire will assist to the highest standards of patient care and blood bank management in order to upgrade the current system.

Email *

drravindran@gmail.com

Name *

Dr M Raveendran

Title/Position *

Senior civil surgeon and Senior Assistant Professor, Blood Centre

Institution/Hospital *

Govt. Mohan Kumaramangalam Medical College, Salem

1. Which blood products play an indispensable role in clinical treatment modalities? *

- [ ] Whole blood
- [x] Red Blood Cells (RBC)
- [ ] Platelets
- [ ] All of the above
- [ ] Other: ____________

2. Which specific blood product(s) are critical for effective inventory management in clinical settings? *

- [ ] Platelets (Shelf-life 5-7 days)
- [ ] RBCs (Shelf-life upto 42 days)
- [ ] Whole blood (Shelf-life upto 35 days)
- [x] All of the above
- [ ] Other: ____________

3. What are the physicians' primary concerns/challenges when sourcing/acquiring blood bags for clinical use? *

- [x] Ensuring the availability of compatible blood products for specific patient needs.
- [ ] Minimizing the risk of transfusion reactions and adverse outcomes.
- [ ] Managing inventory levels to prevent shortages or wastage.
- [ ] All of the above.
- [ ] None of the above.

4. Who should be responsible for checking the expiry date of blood bags during the process of blood transfusion? *

- ( ) Hospital management
- ( ) Blood bank management
- (x) Both
- ( ) Either hospital management or blood bank management
- ( ) Other: ________

5. In clinical practice, does the attending physician specify a preference for fresh blood or ordinary blood when requesting a blood bag? *

- (x) Depends on the patient age, gender and treatment, the product may be either fresh or ordinary
- ( ) Always fresh products
- ( ) Always Ordinary products
- ( ) Age of the product does not matter
- ( ) Other: ________

6. What are the predominant types and frequencies of transfusion reactions observed following platelet transfusions, and what are the associated risk factors, clinical manifestations, and management strategies for each type? *

Protein Allergy

7. What is the comparative incidence and severity of transfusion reactions associated with red blood cell (RBC) transfusions versus platelet transfusions, considering factors such as recipient characteristics, donor factors, and storage duration of blood products? *

More with RBCs

8. What are the potential factors contributing to platelet post-transfusion reactions? *

Contamination

9. Which blood type exhibits the highest frequency among the Indian population, excluding blood type O? *

- ○ Blood type A
- ◉ Blood type B
- ○ Blood type AB
- ○ None of the above
- ○ Other: ______

10. Which blood type exhibits the highest frequency among the global population, excluding blood type O? *

- (x) Blood type A
- ( ) Blood type B
- ( ) Blood type AB
- ( ) None of the above
- ( ) Other: ______

11. What are the primary blood group systems utilised in blood banking practices, with considerations for transfusion compatibility, alloimmunisation risk, and donor-recipient matching? *

- ( ) ABO
- ( ) RhD
- (x) Both ABO and RhD
- ( ) None of the above
- ( ) Other: ______

12. What is the significance of considering blood subtypes in patients undergoing blood transfusions, particularly the potential impacts on transfusion safety, efficacy, and patient outcomes? *

- [ ] Highly improve the patient service level
- [ ] Highly give the advancement in medical treatment
- [x] Preventing any transfusion related reactions without considering the subtypes
- [ ] All of the above
- [ ] Other: ______

13. Ignoring the blood type A1 and A2 variances impact transfusion outcomes and medical complications? *

- ○ Strongly agree
- ○ Agree
- ○ Not sure
- ● Disagree
- ○ Strongly disagree

14. What are the potential medical complications associated with transfusing blood type A2 to patients classified as blood type A, and how do they compare to transfusions with blood type A1? *

- ○ Increased risk of hemolytic reactions due to ABO incompatibility; higher likelihood of alloimmunization against A1 antigen in recipients of A2 blood.
- ◉ Lower risk of hemolytic reactions compared to A1 transfusions; potential for delayed hemolytic reactions due to minor antigenic differences.
- ○ Higher risk of transfusion-related acute lung injury (TRALI) compared to A1 transfusions; increased susceptibility to infectious complications.
- ○ Similar risk of medical complications between A1 and A2 transfusions; no significant differences in clinical outcomes observed.
- ○ Other:

15. Is the classification of blood subtyping within blood type A essential before administering a blood transfusion, to get effective transfusion safety, compatibility, and patient outcomes? *

- ○ Essential and to upgrade the ABO system into A1A2BO system
- ○ Not essential but upgrading the ABO system can provided effective transfusion
- ○ Not essential so no need to upgrade the ABO system
- ○ Essential but upgrades no needed
- ◉ Other: Essential to upgrade, may be to study any delayed reaction occur are not with A1 to A2

16. To what extent is there sufficient awareness among healthcare professionals regarding the implementation of subtyping systems for managing blood inventory levels when individuals know their blood group? *

- ○ High, with comprehensive training programs emphasizing the importance of subtyping and inventory management.
- ○ Moderate, with some awareness of subtyping benefits but potential gaps in understanding its impact on inventory management.
- ○ Low, with limited emphasis on subtyping education, leading to challenges in managing blood inventory effectively.
- ◉ Varied, depending on individual knowledge and practices, resulting in inconsistent implementation of subtyping systems across healthcare settings.
- ○ Other: ____________________

17. What impact does upgrading the blood group system in blood banks have on the quality of service provided, considering factors such as efficiency, accuracy of blood typing, inventory management, and patient outcomes? *

- ◉ Significant, with improved efficiency, accuracy, and patient outcomes observed after implementation.
- ○ Moderate, with some improvements in efficiency and accuracy, but limited impact on patient outcomes.
- ○ Minimal, with little discernible change in service quality despite upgrading the blood group system.
- ○ Varied, depending on factors such as the comprehensiveness of the upgrade and the implementation strategy, resulting in differing levels of enhancement across blood banks.
- ○ Other: ____________________

18. What are the key strategies employed to mitigate potential risks and ensure transfusion safety ? *

- [ ] Implementing advanced screening technologies to detect infectious agents in donated blood.
- [ ] Enhancing donor recruitment efforts to increase the pool of healthy and eligible donors.
- [ ] Utilizing barcode scanning and electronic tracking systems to improve inventory management and traceability.
- [x] All of the above, indicating the need for ongoing upgrades in the blood bank system to ensure optimal patient outcomes.
- [ ] Other: ____________

19. What potential complications arise from strictly adhering to a First-In-First-Out (FIFO) policy in blood banks for blood product distribution, particularly concerning transfusion outcomes and patient safety? *

- [ ] Risk of wastage due to expired blood products, compromising inventory management.
- [ ] Potential mismatch between blood product age and patient needs, affecting transfusion efficacy.
- [ ] Increased likelihood of administering outdated blood products, raising concerns about transfusion safety.
- [x] All of the above.
- [ ] Other: ____________

20. What are the indispensable clinical scenarios where platelet transfusion demonstrates unparalleled efficacy? *

- [x] Severe thrombocytopenia in patients undergoing chemotherapy or bone marrow transplant.
- [ ] Management of bleeding in patients with hematologic disorders like aplastic anemia or leukemia.
- [ ] Surgical procedures involving extensive blood loss or where clotting factors are depleted.
- [ ] All of the above.
- [ ] Other:

21. In cancer patients' treatment, what is the significance of using fresh platelets compared to stored platelets in terms of efficacy, safety, and clinical outcomes? *

- ○ Fresh platelets demonstrate significantly higher efficacy in managing thrombocytopenia during cancer treatment.
- ○ Stored platelets are safer to use due to rigorous screening processes and longer shelf life.
- ◉ Both fresh and stored platelets have similar efficacy and safety profiles in cancer patients undergoing treatment.
- ○ The use of fresh platelets may lead to better clinical outcomes, including reduced bleeding risk and improved transfusion response, compared to stored platelets.
- ○ Other:

22. What is the primary biological rationale for distinguishing between Single Donor Platelets (SDP) and Random Donor Platelets (RDP) in blood transfusion practices? *

- ○ SDP and RDP differ primarily in their Rh factor compatibility, affecting transfusion safety and efficacy.
- ○ Rh factor plays a negligible role in the classification of SDP and RDP, with other factors such as shelf life being more significant.
- ○ Both SDP and RDP are classified based on Rh factor compatibility, ensuring appropriate matching for transfusion recipients.
- ◉ The classification of SDP and RDP is unrelated to Rh factor, instead focusing on donor screening and processing methods.
- ○ Other: ______

23. What are the primary reasons underlying the distinct classification of Single Donor Platelets (SDP) and Random Donor Platelets (RDP) in blood bank management systems? *

- ○ A) SDP and RDP have different storage requirements, affecting inventory management and shelf life.
- ○ B) The classification ensures appropriate matching based on donor-recipient compatibility, including Rh factor.
- ◉ C) SDP and RDP vary in terms of availability and cost-effectiveness, impacting resource allocation.
- ○ D) Both A and B.
- ○ Other: ______

24. In the treatment of oncology patients, which is preferable between platelet apheresis and platelet concentrate in terms of efficacy, safety, and overall patient outcomes? *

- (●) Platelet apheresis, due to its ability to provide a higher concentration of platelets per transfusion.
- ( ) Platelet concentrate, as it is more readily available and less labor-intensive compared to apheresis.
- ( ) Both platelet apheresis and platelet concentrate demonstrate similar efficacy and safety profiles in oncology patients.
- ( ) The preference between platelet apheresis and platelet concentrate depends on individual patient factors and clinical scenarios.
- ( ) Other:

25. What is the principal objective of blood banks in the healthcare systems? *

- [x] To ensure an adequate supply of safe and compatible blood products for transfusion.
- [ ] To conduct research on blood-related diseases and disorders.
- [ ] To provide educational programs for healthcare professionals on blood transfusion practices.
- [ ] To manage blood donations and organize blood drives within the community.
- [ ] Other:

26. What should blood banks prioritise medical objectives when allocating blood products to patients? *

- [ ] Ensuring compatibility between donor blood and recipient to prevent adverse reactions.
- [ ] Minimizing the risk of transfusion-transmitted infections through stringent screening procedures.
- [ ] Addressing the immediate medical needs of patients based on their clinical condition and urgency.
- [x] All of the above.
- [ ] Other: ____________

27. Which is the most difficult process of blood bank management either allocating to patients or ordering process to maintain inventory levels? *

- ( ) Blood products allocation to patients, ensuring appropriate matching and timely delivery.
- ( ) Ordering process to maintain inventory levels, balancing supply and demand while minimizing wastage.
- ( ) Both allocation to patients and ordering process pose equal challenges in blood bank management.
- (•) The difficulty varies depending on factors such as facility size, patient population, and resource availability.
- ( ) Other: ____________

28. What are the key factors the blood bank should follow to reduce shortages? *

- [ ] Implementing efficient inventory management systems to monitor and optimize blood product levels.
- [x] Developing effective donor recruitment strategies to ensure a consistent and sufficient blood donor pool.
- [ ] Implementing transfusion protocols that prioritize appropriate and judicious use of blood products.
- [ ] Maximizing utilization efficiency by minimizing wastage and optimizing allocation to meet patient needs.
- [ ] Other:

29. What role does cross-matching play in minimising blood shortages in the context of blood transfusion? *

- ( ) Cross-matching ensures compatibility between donor blood and recipient, reducing the risk of transfusion reactions and wastage.
- ( ) Cross-matching helps identify suitable blood donors, thus increasing the available blood supply and minimizing shortages.
- ( ) Cross-matching optimizes blood utilization by ensuring that blood products are used only when necessary, thereby conserving resources.
- ( ) Cross-matching streamlines the transfusion process, leading to faster turnaround times and increased efficiency in addressing blood shortages.
- (•) Other: -

30. What is the significance of blood bank managers regarding the importance of knowing the treatment of patients before issuing blood bags for transfusion? *

- ○ It ensures that the appropriate blood products are selected based on the patient's specific medical condition and treatment regimen.
- ○ It helps minimize the risk of adverse reactions or complications resulting from incompatible blood transfusions.
- ○ It allows blood bank managers to track trends in blood product usage and tailor inventory management strategies accordingly.
- ◉ All of the above.
- ○ Other: ____________

31. How does adhering to a First-In-First-Out (FIFO) policy for allocating platelet products, without consideration of the patient's treatment, impact the occurrence of transfusion reactions and the improvement of CCI? *

- [ ] A) It increases the risk of platelet transfusion reactions due to potential mismatches between donor blood and recipient.
- [ ] B) It may lead to a lack of improvement in the Corrected Count Increment (CCI) as the selected blood products may not be suitable for the patient's treatment.
- [ ] C) Both A and B.
- [x] D) Adherence to FIFO policy does not impact the occurrence of transfusion reactions or the improvement of CCI.
- [ ] Other: ____________

This form was created inside of Vellore Institute of Technology.

Google Forms

Research Questions

With the help of these questions, blood banking and transfusion treatment will advance! Explore the key strategies employed by blood banks to mitigate risks and ensure transfusion safety, and discover the latest advancements in the field. With a focus on upgrading the current blood bank system, this questionnaire will assist to the highest standards of patient care and blood bank management in order to upgrade the current system.

Email *

megalai.mbbs@gmail.com

Name *

Dr.B.Manimegalai

Title/Position *

M.S.OBG

Institution/Hospital *

Tagore medical college and Hospital

1. Which blood products play an indispensable role in clinical treatment modalities? *

- [ ] Whole blood
- [ ] Red Blood Cells (RBC)
- [ ] Platelets
- [x] All of the above
- [ ] Other: ______

2. Which specific blood product(s) are critical for effective inventory management in clinical settings? *

- [ ] Platelets (Shelf-life 5-7 days)
- [ ] RBCs (Shelf-life upto 42 days)
- [ ] Whole blood (Shelf-life upto 35 days)
- [x] All of the above
- [ ] Other: ______

3. What are the physicians' primary concerns/challenges when sourcing/acquiring blood bags for clinical use? *

- [ ] Ensuring the availability of compatible blood products for specific patient needs.
- [ ] Minimizing the risk of transfusion reactions and adverse outcomes.
- [ ] Managing inventory levels to prevent shortages or wastage.
- [x] All of the above.
- [ ] None of the above.

4. Who should be responsible for checking the expiry date of blood bags during the process of blood transfusion? *

- ( ) Hospital management
- ( ) Blood bank management
- (x) Both
- ( ) Either hospital management or blood bank management
- ( ) Other: ____

5. In clinical practice, does the attending physician specify a preference for fresh blood or ordinary blood when requesting a blood bag? *

- ( ) Depends on the patient age, gender and treatment, the product may be either fresh or ordinary
- ( ) Always fresh products
- ( ) Always Ordinary products
- ( ) Age of the product does not matter
- (x) Other: depends on the gender, age of the patient and treatment the product age may differ.

6. What are the predominant types and frequencies of transfusion reactions observed following platelet transfusions, and what are the associated risk factors, clinical manifestations, and management strategies for each type? *

Early reaction, Risk factors are known previous allergic reaction to blood and blood products, clinical features are Dyspnoea, urticaria,angioedema.Management is immediately to stop transfusion then to give steroids.

7. What is the comparative incidence and severity of transfusion reactions associated with red blood cell (RBC) transfusions versus platelet transfusions, considering factors such as recipient characteristics, donor factors, and storage duration of blood products? *

Incidence same for RBC and Platelets because ABO incompatibility and RH incompatibility is more common. Also storage of longer duration of blood products will cause lysis of cells and MOD.

8. What are the potential factors contributing to platelet post-transfusion reactions? *

Diabetes like hyperglycemia and fever,bleeding, heparin administration,lymphocytotoxic antibody reaction,infections.

9. Which blood type exhibits the highest frequency among the Indian population, excluding blood type O? *

- ○ Blood type A
- ◉ Blood type B
- ○ Blood type AB
- ○ None of the above
- ○ Other: ______

10. Which blood type exhibits the highest frequency among the global population, excluding blood type O? *

- (●) Blood type A
- ( ) Blood type B
- ( ) Blood type AB
- ( ) None of the above
- ( ) Other:

11. What are the primary blood group systems utilised in blood banking practices, with considerations for transfusion compatibility, alloimmunisation risk, and donor-recipient matching? *

- ( ) ABO
- ( ) RhD
- (●) Both ABO and RhD
- ( ) None of the above
- ( ) Other:

12. What is the significance of considering blood subtypes in patients undergoing blood transfusions, particularly the potential impacts on transfusion safety, efficacy, and patient outcomes? *

- [ ] Highly improve the patient service level
- [ ] Highly give the advancement in medical treatment
- [ ] Preventing any transfusion related reactions without considering the subtypes
- [x] All of the above
- [ ] Other: ______

13. Ignoring the blood type A1 and A2 variances impact transfusion outcomes and medical complications? *

- ( ) Strongly agree
- (•) Agree
- ( ) Not sure
- ( ) Disagree
- ( ) Strongly disagree

14. What are the potential medical complications associated with transfusing blood type A2 to patients classified as blood type A, and how do they compare to transfusions with blood type A1? *

- (selected) Increased risk of hemolytic reactions due to ABO incompatibility; higher likelihood of alloimmunization against A1 antigen in recipients of A2 blood.
- Lower risk of hemolytic reactions compared to A1 transfusions; potential for delayed hemolytic reactions due to minor antigenic differences.
- Higher risk of transfusion-related acute lung injury (TRALI) compared to A1 transfusions; increased susceptibility to infectious complications.
- Similar risk of medical complications between A1 and A2 transfusions; no significant differences in clinical outcomes observed.
- Other:

15. Is the classification of blood subtyping within blood type A essential before administering a blood transfusion, to get effective transfusion safety, compatibility, and patient outcomes? *

- Essential and to upgrade the ABO system into A1A2BO system
- Not essential but upgrading the ABO system can provided effective transfusion
- Not essential so no need to upgrade the ABO system
- Essential but upgrades no needed
- (selected) Other:

Yes. a person receives an incompatible blood type during a transfusion, the immune system of the person receiving the transfusion goes into a massive activation and attacks the invading blood. Clotting may occur, resulting in complications such as kidney failure, circulatory failure, shock, or possibly, even death.

16. To what extent is there sufficient awareness among healthcare professionals regarding the implementation of subtyping systems for managing blood inventory levels when individuals know their blood group? *

- ○ High, with comprehensive training programs emphasizing the importance of subtyping and inventory management.
- ◉ Moderate, with some awareness of subtyping benefits but potential gaps in understanding its impact on inventory management.
- ○ Low, with limited emphasis on subtyping education, leading to challenges in managing blood inventory effectively.
- ○ Varied, depending on individual knowledge and practices, resulting in inconsistent implementation of subtyping systems across healthcare settings.
- ○ Other: ____________

17. What impact does upgrading the blood group system in blood banks have on the quality of service provided, considering factors such as efficiency, accuracy of blood typing, inventory management, and patient outcomes? *

- ◉ Significant, with improved efficiency, accuracy, and patient outcomes observed after implementation.
- ○ Moderate, with some improvements in efficiency and accuracy, but limited impact on patient outcomes.
- ○ Minimal, with little discernible change in service quality despite upgrading the blood group system.
- ○ Varied, depending on factors such as the comprehensiveness of the upgrade and the implementation strategy, resulting in differing levels of enhancement across blood banks.
- ○ Other: ____________

18. What are the key strategies employed to mitigate potential risks and ensure transfusion safety ? *

- [ ] Implementing advanced screening technologies to detect infectious agents in donated blood.
- [ ] Enhancing donor recruitment efforts to increase the pool of healthy and eligible donors.
- [ ] Utilizing barcode scanning and electronic tracking systems to improve inventory management and traceability.
- [x] All of the above, indicating the need for ongoing upgrades in the blood bank system to ensure optimal patient outcomes.
- [ ] Other: ____________

19. What potential complications arise from strictly adhering to a First-In-First-Out (FIFO) policy in blood banks for blood product distribution, particularly concerning transfusion outcomes and patient safety? *

- [ ] Risk of wastage due to expired blood products, compromising inventory management.
- [x] Potential mismatch between blood product age and patient needs, affecting transfusion efficacy.
- [ ] Increased likelihood of administering outdated blood products, raising concerns about transfusion safety.
- [ ] All of the above.
- [ ] Other: ____________

20. What are the indispensable clinical scenarios where platelet transfusion demonstrates unparalleled efficacy? *

- [ ] Severe thrombocytopenia in patients undergoing chemotherapy or bone marrow transplant.
- [ ] Management of bleeding in patients with hematologic disorders like aplastic anemia or leukemia.
- [ ] Surgical procedures involving extensive blood loss or where clotting factors are depleted.
- [x] All of the above.
- [ ] Other:

21. In cancer patients' treatment, what is the significance of using fresh platelets compared to stored platelets in terms of efficacy, safety, and clinical outcomes? *

- (●) Fresh platelets demonstrate significantly higher efficacy in managing thrombocytopenia during cancer treatment.
- ( ) Stored platelets are safer to use due to rigorous screening processes and longer shelf life.
- ( ) Both fresh and stored platelets have similar efficacy and safety profiles in cancer patients undergoing treatment.
- ( ) The use of fresh platelets may lead to better clinical outcomes, including reduced bleeding risk and improved transfusion response, compared to stored platelets.
- ( ) Other:

22. What is the primary biological rationale for distinguishing between Single Donor Platelets (SDP) and Random Donor Platelets (RDP) in blood transfusion practices? *

- ( ) SDP and RDP differ primarily in their Rh factor compatibility, affecting transfusion safety and efficacy.
- ( ) Rh factor plays a negligible role in the classification of SDP and RDP, with other factors such as shelf life being more significant.
- (●) Both SDP and RDP are classified based on Rh factor compatibility, ensuring appropriate matching for transfusion recipients.
- ( ) The classification of SDP and RDP is unrelated to Rh factor, instead focusing on donor screening and processing methods.
- ( ) Other: ____________

23. What are the primary reasons underlying the distinct classification of Single Donor Platelets (SDP) and Random Donor Platelets (RDP) in blood bank management systems? *

- ( ) A) SDP and RDP have different storage requirements, affecting inventory management and shelf life.
- ( ) B) The classification ensures appropriate matching based on donor-recipient compatibility, including Rh factor.
- ( ) C) SDP and RDP vary in terms of availability and cost-effectiveness, impacting resource allocation.
- (●) D) Both A and B.
- ( ) Other: ____________

24. In the treatment of oncology patients, which is preferable between platelet apheresis and platelet concentrate in terms of efficacy, safety, and overall patient outcomes? *

- ○ Platelet apheresis, due to its ability to provide a higher concentration of platelets per transfusion.
- ○ Platelet concentrate, as it is more readily available and less labor-intensive compared to apheresis.
- ○ Both platelet apheresis and platelet concentrate demonstrate similar efficacy and safety profiles in oncology patients.
- ◉ The preference between platelet apheresis and platelet concentrate depends on individual patient factors and clinical scenarios.
- ○ Other: ____________________

25. What is the principal objective of blood banks in the healthcare systems? *

- [x] To ensure an adequate supply of safe and compatible blood products for transfusion.
- [ ] To conduct research on blood-related diseases and disorders.
- [ ] To provide educational programs for healthcare professionals on blood transfusion practices.
- [ ] To manage blood donations and organize blood drives within the community.
- [ ] Other: ____________________

26. What should blood banks prioritise medical objectives when allocating blood products to patients? *

- [ ] Ensuring compatibility between donor blood and recipient to prevent adverse reactions.
- [ ] Minimizing the risk of transfusion-transmitted infections through stringent screening procedures.
- [ ] Addressing the immediate medical needs of patients based on their clinical condition and urgency.
- [x] All of the above.
- [ ] Other:

27. Which is the most difficult process of blood bank management either allocating to patients or ordering process to maintain inventory levels? *

- ( ) Blood products allocation to patients, ensuring appropriate matching and timely delivery.
- ( ) Ordering process to maintain inventory levels, balancing supply and demand while minimizing wastage.
- (•) Both allocation to patients and ordering process pose equal challenges in blood bank management.
- ( ) The difficulty varies depending on factors such as facility size, patient population, and resource availability.
- ( ) Other:

28. What are the key factors the blood bank should follow to reduce shortages? *

- [x] Implementing efficient inventory management systems to monitor and optimize blood product levels.
- [x] Developing effective donor recruitment strategies to ensure a consistent and sufficient blood donor pool.
- [x] Implementing transfusion protocols that prioritize appropriate and judicious use of blood products.
- [x] Maximizing utilization efficiency by minimizing wastage and optimizing allocation to meet patient needs.
- [ ] Other:

29. What role does cross-matching play in minimising blood shortages in the context of blood transfusion? *

- (●) Cross-matching ensures compatibility between donor blood and recipient, reducing the risk of transfusion reactions and wastage.
- ( ) Cross-matching helps identify suitable blood donors, thus increasing the available blood supply and minimizing shortages.
- ( ) Cross-matching optimizes blood utilization by ensuring that blood products are used only when necessary, thereby conserving resources.
- ( ) Cross-matching streamlines the transfusion process, leading to faster turnaround times and increased efficiency in addressing blood shortages.
- ( ) Other:

30. What is the significance of blood bank managers regarding the importance of knowing the treatment of patients before issuing blood bags for transfusion? *

- ( ) It ensures that the appropriate blood products are selected based on the patient's specific medical condition and treatment regimen.
- ( ) It helps minimize the risk of adverse reactions or complications resulting from incompatible blood transfusions.
- ( ) It allows blood bank managers to track trends in blood product usage and tailor inventory management strategies accordingly.
- (•) All of the above.
- ( ) Other:

31. How does adhering to a First-In-First-Out (FIFO) policy for allocating platelet products, without consideration of the patient's treatment, impact the occurrence of transfusion reactions and the improvement of CCI? *

- [ ] A) It increases the risk of platelet transfusion reactions due to potential mismatches between donor blood and recipient.
- [ ] B) It may lead to a lack of improvement in the Corrected Count Increment (CCI) as the selected blood products may not be suitable for the patient's treatment.
- [ ] C) Both A and B.
- [x] D) Adherence to FIFO policy does not impact the occurrence of transfusion reactions or the improvement of CCI.
- [x] Other: I suggest that FIFO policy is not applicable in pregnancy and children. Both the groups are transfused by fresh blood and blood products.

This form was created inside of Vellore Institute of Technology.

Google Forms

Research Questions

With the help of these questions, blood banking and transfusion treatment will advance! Explore the key strategies employed by blood banks to mitigate risks and ensure transfusion safety, and discover the latest advancements in the field. With a focus on upgrading the current blood bank system, this questionnaire will assist to the highest standards of patient care and blood bank management in order to upgrade the current system.

Email *

ramspriya08@gmail.com

Name *

V.Ramasamy

Title/Position *

Technical supervisor

Institution/Hospital *

Govt.M.K.M.C.Hospital, Salem

1. Which blood products play an indispensable role in clinical treatment modalities? *

- [ ] Whole blood
- [ ] Red Blood Cells (RBC)
- [ ] Platelets
- [x] All of the above
- [ ] Other: ____________

2. Which specific blood product(s) are critical for effective inventory management in clinical settings? *

- [ ] Platelets (Shelf-life 5-7 days)
- [ ] RBCs (Shelf-life upto 42 days)
- [ ] Whole blood (Shelf-life upto 35 days)
- [x] All of the above
- [ ] Other: ____________

3. What are the physicians' primary concerns/challenges when sourcing/acquiring blood bags for clinical use? *

- [ ] Ensuring the availability of compatible blood products for specific patient needs.
- [ ] Minimizing the risk of transfusion reactions and adverse outcomes.
- [ ] Managing inventory levels to prevent shortages or wastage.
- [x] All of the above.
- [ ] None of the above.

4. Who should be responsible for checking the expiry date of blood bags during the process of blood transfusion? *

- ○ Hospital management
- ○ Blood bank management
- ○ Both
- ○ Either hospital management or blood bank management
- ◉ Other: Lab Technician

5. In clinical practice, does the attending physician specify a preference for fresh blood or ordinary blood when requesting a blood bag? *

- ◉ Depends on the patient age, gender and treatment, the product may be either fresh or ordinary
- ○ Always fresh products
- ○ Always Ordinary products
- ○ Age of the product does not matter
- ○ Other:

6. What are the predominant types and frequencies of transfusion reactions observed following platelet transfusions, and what are the associated risk factors, clinical manifestations, and management strategies for each type? *

Transfusion reactions may be seen in up to 1% of transfusions. Transfusion reactions can range from mild to life-threatening events. Transfusion reactions can rarely be fatal.

7. What is the comparative incidence and severity of transfusion reactions associated with red blood cell (RBC) transfusions versus platelet transfusions, considering factors such as recipient characteristics, donor factors, and storage duration of blood products? *

Febrile non-hemolytic transfusion reaction

8. What are the potential factors contributing to platelet post-transfusion reactions? *

IgE and IgG antibodies in the recipient against plasma proteins in the transfused blood component,

9. Which blood type exhibits the highest frequency among the Indian population, excluding blood type O? *

- ○ Blood type A
- ◉ Blood type B
- ○ Blood type AB
- ○ None of the above
- ○ Other:

10. Which blood type exhibits the highest frequency among the global population, excluding blood type O? *

- ○ Blood type A
- ◉ Blood type B
- ○ Blood type AB
- ○ None of the above
- ○ Other: ____________

11. What are the primary blood group systems utilised in blood banking practices, with considerations for transfusion compatibility, alloimmunisation risk, and donor-recipient matching? *

- ○ ABO
- ○ RhD
- ◉ Both ABO and RhD
- ○ None of the above
- ○ Other: ____________

12. What is the significance of considering blood subtypes in patients undergoing blood transfusions, particularly the potential impacts on transfusion safety, efficacy, and patient outcomes? *

- [ ] Highly improve the patient service level
- [ ] Highly give the advancement in medical treatment
- [ ] Preventing any transfusion related reactions without considering the subtypes
- [x] All of the above
- [ ] Other: ____________

13. Ignoring the blood type A1 and A2 variances impact transfusion outcomes and medical complications? *

- ( ) Strongly agree
- ( ) Agree
- ( ) Not sure
- (•) Disagree
- ( ) Strongly disagree

14. What are the potential medical complications associated with transfusing blood type A2 to patients classified as blood type A, and how do they compare to transfusions with blood type A1? *

- ( ) Increased risk of hemolytic reactions due to ABO incompatibility; higher likelihood of alloimmunization against A1 antigen in recipients of A2 blood.
- (•) Lower risk of hemolytic reactions compared to A1 transfusions; potential for delayed hemolytic reactions due to minor antigenic differences.
- ( ) Higher risk of transfusion-related acute lung injury (TRALI) compared to A1 transfusions; increased susceptibility to infectious complications.
- ( ) Similar risk of medical complications between A1 and A2 transfusions; no significant differences in clinical outcomes observed.
- ( ) Other: ____________

15. Is the classification of blood subtyping within blood type A essential before administering a blood transfusion, to get effective transfusion safety, compatibility, and patient outcomes? *

- ( ) Essential and to upgrade the ABO system into A1A2BO system
- ( ) Not essential but upgrading the ABO system can provided effective transfusion
- (•) Not essential so no need to upgrade the ABO system
- ( ) Essential but upgrades no needed
- ( ) Other: ____________

16. To what extent is there sufficient awareness among healthcare professionals regarding the implementation of subtyping systems for managing blood inventory levels when individuals know their blood group? *

- ○ High, with comprehensive training programs emphasizing the importance of subtyping and inventory management.
- ○ Moderate, with some awareness of subtyping benefits but potential gaps in understanding its impact on inventory management.
- ◉ Low, with limited emphasis on subtyping education, leading to challenges in managing blood inventory effectively.
- ○ Varied, depending on individual knowledge and practices, resulting in inconsistent implementation of subtyping systems across healthcare settings.
- ○ Other: ______________________

17. What impact does upgrading the blood group system in blood banks have on the quality of service provided, considering factors such as efficiency, accuracy of blood typing, inventory management, and patient outcomes? *

- ○ Significant, with improved efficiency, accuracy, and patient outcomes observed after implementation.
- ○ Moderate, with some improvements in efficiency and accuracy, but limited impact on patient outcomes.
- ◉ Minimal, with little discernible change in service quality despite upgrading the blood group system.
- ○ Varied, depending on factors such as the comprehensiveness of the upgrade and the implementation strategy, resulting in differing levels of enhancement across blood banks.
- ○ Other: ______________________

18. What are the key strategies employed to mitigate potential risks and ensure transfusion safety ? *

- [ ] Implementing advanced screening technologies to detect infectious agents in donated blood.
- [ ] Enhancing donor recruitment efforts to increase the pool of healthy and eligible donors.
- [ ] Utilizing barcode scanning and electronic tracking systems to improve inventory management and traceability.
- [x] All of the above, indicating the need for ongoing upgrades in the blood bank system to ensure optimal patient outcomes.
- [ ] Other: ____________________

19. What potential complications arise from strictly adhering to a First-In-First-Out (FIFO) policy in blood banks for blood product distribution, particularly concerning transfusion outcomes and patient safety? *

- [x] Risk of wastage due to expired blood products, compromising inventory management.
- [ ] Potential mismatch between blood product age and patient needs, affecting transfusion efficacy.
- [ ] Increased likelihood of administering outdated blood products, raising concerns about transfusion safety.
- [ ] All of the above.
- [ ] Other: ____________________

20. What are the indispensable clinical scenarios where platelet transfusion demonstrates unparalleled efficacy? *

- [ ] Severe thrombocytopenia in patients undergoing chemotherapy or bone marrow transplant.
- [ ] Management of bleeding in patients with hematologic disorders like aplastic anemia or leukemia.
- [ ] Surgical procedures involving extensive blood loss or where clotting factors are depleted.
- [x] All of the above.
- [ ] Other: ____________

21. In cancer patients' treatment, what is the significance of using fresh platelets compared to stored platelets in terms of efficacy, safety, and clinical outcomes? *

- ( ) Fresh platelets demonstrate significantly higher efficacy in managing thrombocytopenia during cancer treatment.
- ( ) Stored platelets are safer to use due to rigorous screening processes and longer shelf life.
- (•) Both fresh and stored platelets have similar efficacy and safety profiles in cancer patients undergoing treatment.
- ( ) The use of fresh platelets may lead to better clinical outcomes, including reduced bleeding risk and improved transfusion response, compared to stored platelets.
- ( ) Other: ____________

22. What is the primary biological rationale for distinguishing between Single Donor Platelets (SDP) and Random Donor Platelets (RDP) in blood transfusion practices? *

- ○ SDP and RDP differ primarily in their Rh factor compatibility, affecting transfusion safety and efficacy.
- ◉ Rh factor plays a negligible role in the classification of SDP and RDP, with other factors such as shelf life being more significant.
- ○ Both SDP and RDP are classified based on Rh factor compatibility, ensuring appropriate matching for transfusion recipients.
- ○ The classification of SDP and RDP is unrelated to Rh factor, instead focusing on donor screening and processing methods.
- ○ Other: ____________________

23. What are the primary reasons underlying the distinct classification of Single Donor Platelets (SDP) and Random Donor Platelets (RDP) in blood bank management systems? *

- ○ A) SDP and RDP have different storage requirements, affecting inventory management and shelf life.
- ○ B) The classification ensures appropriate matching based on donor-recipient compatibility, including Rh factor.
- ◉ C) SDP and RDP vary in terms of availability and cost-effectiveness, impacting resource allocation.
- ○ D) Both A and B.
- ○ Other: ____________________

24. In the treatment of oncology patients, which is preferable between platelet apheresis and platelet concentrate in terms of efficacy, safety, and overall patient outcomes? *

- (●) Platelet apheresis, due to its ability to provide a higher concentration of platelets per transfusion.
- ( ) Platelet concentrate, as it is more readily available and less labor-intensive compared to apheresis.
- ( ) Both platelet apheresis and platelet concentrate demonstrate similar efficacy and safety profiles in oncology patients.
- ( ) The preference between platelet apheresis and platelet concentrate depends on individual patient factors and clinical scenarios.
- ( ) Other: ____________

25. What is the principal objective of blood banks in the healthcare systems? *

- [x] To ensure an adequate supply of safe and compatible blood products for transfusion.
- [ ] To conduct research on blood-related diseases and disorders.
- [ ] To provide educational programs for healthcare professionals on blood transfusion practices.
- [ ] To manage blood donations and organize blood drives within the community.
- [ ] Other: ____________

26. What should blood banks prioritise medical objectives when allocating blood products to patients? *

- [x] Ensuring compatibility between donor blood and recipient to prevent adverse reactions.
- [ ] Minimizing the risk of transfusion-transmitted infections through stringent screening procedures.
- [ ] Addressing the immediate medical needs of patients based on their clinical condition and urgency.
- [ ] All of the above.
- [ ] Other:

27. Which is the most difficult process of blood bank management either allocating to patients or ordering process to maintain inventory levels? *

- ○ Blood products allocation to patients, ensuring appropriate matching and timely delivery.
- ○ Ordering process to maintain inventory levels, balancing supply and demand while minimizing wastage.
- ◉ Both allocation to patients and ordering process pose equal challenges in blood bank management.
- ○ The difficulty varies depending on factors such as facility size, patient population, and resource availability.
- ○ Other:

28. What are the key factors the blood bank should follow to reduce shortages? *

- [x] Implementing efficient inventory management systems to monitor and optimize blood product levels.
- [ ] Developing effective donor recruitment strategies to ensure a consistent and sufficient blood donor pool.
- [ ] Implementing transfusion protocols that prioritize appropriate and judicious use of blood products.
- [ ] Maximizing utilization efficiency by minimizing wastage and optimizing allocation to meet patient needs.
- [ ] Other:

29. What role does cross-matching play in minimising blood shortages in the context of blood transfusion? *

- (●) Cross-matching ensures compatibility between donor blood and recipient, reducing the risk of transfusion reactions and wastage.
- ( ) Cross-matching helps identify suitable blood donors, thus increasing the available blood supply and minimizing shortages.
- ( ) Cross-matching optimizes blood utilization by ensuring that blood products are used only when necessary, thereby conserving resources.
- ( ) Cross-matching streamlines the transfusion process, leading to faster turnaround times and increased efficiency in addressing blood shortages.
- ( ) Other:

30. What is the significance of blood bank managers regarding the importance of knowing the treatment of patients before issuing blood bags for transfusion? *

- [ ] It ensures that the appropriate blood products are selected based on the patient's specific medical condition and treatment regimen.
- [x] It helps minimize the risk of adverse reactions or complications resulting from incompatible blood transfusions.
- [ ] It allows blood bank managers to track trends in blood product usage and tailor inventory management strategies accordingly.
- [ ] All of the above.
- [ ] Other:

31. How does adhering to a First-In-First-Out (FIFO) policy for allocating platelet products, without consideration of the patient's treatment, impact the occurrence of transfusion reactions and the improvement of CCI? *

- [ ] A) It increases the risk of platelet transfusion reactions due to potential mismatches between donor blood and recipient.
- [ ] B) It may lead to a lack of improvement in the Corrected Count Increment (CCI) as the selected blood products may not be suitable for the patient's treatment.
- [x] C) Both A and B.
- [ ] D) Adherence to FIFO policy does not impact the occurrence of transfusion reactions or the improvement of CCI.
- [ ] Other:

This form was created inside of Vellore Institute of Technology.

Google Forms

Expert Report

Name : Dr. M. Raveendran

Title/Position : Senior Civil Surgeon and Senior Assistant Professor, Blood Centre

Institution/Hospital : Govt. Mohan Kumaramangalam Medical College, Salem

Years of experience in the field : 20 years

Specialization with blood bank management : 20 years

Any additional relevant credentials or qualifications : District blood transfusion officer

Declaration

I, Dr. M. Raveendran, hereby acknowledge my participation in the research questionnaire conducted by Chithraponnu R (Research scholar) and Dr. Umamaheswari S (Supervisor). I confirm that the information provided in response to the questionnaire is accurate to my knowledge. I understand that my insights will be used for research purposes and hereby consent to their inclusion in the study.

22/4/24

(DR. M. RAVEENDRAN)

Signature with date

Expert Report

**Name**	**: Mr. V. Ramasamy**
**Title/Position**	**: Technical Supervisor, Blood Bank**
**Institution/Hospital**	**: Govt. M.K.M.C. Hospital, Salem**
**Years of experience in the field**	**: 15 years**
**Specialization in blood bank management**	**: 15 years**
**Any additional relevant qualifications**	**: M.Sc.,(Micro), B.Sc., (MLT), CMLT, PGDCA**

Declaration

I, **Mr. V. Ramasamy**, hereby acknowledge my participation in the research questionnaire conducted by Chithraponnu R (Research scholar) and Dr. Umamaheswari S (Supervisor). I confirm that the information provided in response to the questionnaire is accurate to my knowledge. I understand that my insights will be used for research purposes and hereby consent to their inclusion in the study.

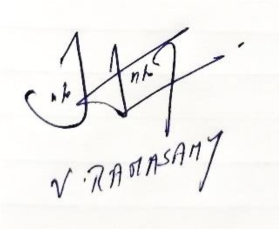

Signature with date

Expert Report

**Name**	**: Dr.B.Manimegalai**
**Title/Position**	**: Assistant surgeon**
**Institution/Hospital**	**: Tagore Medical College and Hospital, Chennai**
**Years of experience in the field**	**: 6 years**
**Specialization with blood bank products**	**: 6 years**
**Any additional relevant credentials or qualifications**	**: M.S.OBG, D.Diab.,**

Declaration

I, **Dr.B.Manimegalai**, hereby acknowledge my participation in the research questionnaire conducted by Chithraponnu R (Research scholar) and Dr. Umamaheswari S (Supervisor). I confirm that the information provided in response to the questionnaire is accurate to my knowledge. I understand that my insights will be used for research purposes and hereby consent to their inclusion in the study.

B. Manimegalai
8.4.2024

Signature with date
